# Tenascin-C can Serve as an Indicator for the Immunosuppressive Microenvironment of Diffuse Low-Grade Gliomas

**DOI:** 10.3389/fimmu.2022.824586

**Published:** 2022-03-16

**Authors:** Po Zhang, Guohao Liu, Jinyang Hu, Sui Chen, Baofeng Wang, Peng Peng, Xingjiang Yu, Dongsheng Guo

**Affiliations:** ^1^ Department of Neurosurgery, Tongji Hospital, Tongji Medical College, Huazhong University of Science and Technology, Wuhan, China; ^2^ Department of Neurosurgery, The People’s Hospital of China Three Gorges University, Yichang, China; ^3^ Department of Histology and Embryology, School of Basic Medicine, Tongji Medical College, Huazhong University of Science and Technology, Wuhan, China

**Keywords:** diffuse low-grade gliomas, tumor immune microenvironment, immunosuppressive, immunotherapy, tenascin-C

## Abstract

**Purpose:**

The development and progression of glioma are associated with the tumor immune microenvironment. Diffuse low-grade gliomas (LGGs) with higher immunosuppressive microenvironment tend to have a poorer prognosis. The study aimed to find a biological marker that can reflect the tumor immune microenvironment status and predict prognosis of LGGs.

**Methods:**

The target gene tenascin-C (TNC) was obtained by screening the Chinese Glioma Genome Atlas (CGGA) and the Cancer Genome Atlas (TCGA) databases. Then samples of LGGs were collected for experimental verification with immunohistochemistry, immunofluorescence, immunoblotting, quantitative real-time PCR. ELISA was employed to determine the content of TNC in serum and examine its relationship with the tumor immune microenvironment. Eventually, the sensitivity of immunotherapy was predicted on the basis of the content of TNC in LGGs.

**Results:**

In the high-TNC subgroup, the infiltration of immunosuppressive cells was increased (MDSC: r=0.4721, Treg: r=0.3154, etc.), and immune effector cells were decreased [NKT, γδT, etc. (p<0.05)], immunosuppressive factors were elevated [TGF-β, IL10, *etc.* (*p*<0.05)], immunostimulatory factors, such as NKG2D, dropped (*p*<0.05), hypoxia scores increased (*p*<0.001), and less benefit from immunotherapy (*p*<0.05). Serum TNC level could be used to assess the status of tumor immune microenvironment in patients with grade II (AUC=0.8571; 95% CI: 0.6541-1.06) and grade III (AUC=0.8333; 95% CI: 0.6334-1.033) glioma.

**Conclusions:**

Our data suggested that TNC could serve as an indicator for the immunosuppressive microenvironment status and the prognosis of LGGs. Moreover, it could also act as a predictor for the effect of immunotherapy on LGG patients.

## Introduction

Glioma represents the most common primary central nervous system tumor with high morbidity and mortality (1, 2). Against the criteria developed by the World Health Organization (WHO), gliomas are histopathologically classified into grades I to IV (3). Grade I glioma is considered benign and can be completely removed by surgery, rarely evolving into higher-grade lesions (4). On the other hand, grade II or III gliomas, collectively known as diffuse low-grade gliomas (LGGs), are aggressive and can recur after resection or directly progress to higher-grade lesions (5, 6). Furthermore, grade IV glioma (glioblastoma, GBM) is the most aggressive with the most unfavorable prognosis (7, 8). The prognosis of LGGs varies greatly and LGG patients with isocitrate dehydrogenase 1 and 2 genes (IDH 1/2) mutations and chromosome arms 1p and 19q (1p/19q) co-deletion tend to have a more favorable prognosis (4, 9). Unfortunately, most LGGs without an IDH mutation were molecularly and clinically mimic glioblastoma (9). In this study, we aimed to find a sensitive indicator (like IDH and 1p/19q) that can assist in the prediction of the progression of LGGs.

In recent years, immunotherapies, especially treatments with immune checkpoint inhibitors, which evoked anti-tumor immune responses to inhibit tumor immune escape, have shown a promise in the treatment of non-central nervous system tumors such as melanoma and colorectal cancer (10). Nonetheless, due to the unique immune microenvironment and strong immunosuppressive status of glioma, the effect of immunotherapies on the tumor remains unsatisfactory (11). Studies have found that the immune microenvironment of LGGs is linked to the IDH mutations, which can roughly predict the prognosis of patients and the efficacy of immunotherapy (12). Therefore, we were led to speculate that a subgroup in LGGs patients might be sensitive to immunotherapy and the corresponding markers can help to identify the subgroup. In this study, we tried to find an indicator that can directly reflect the immune microenvironment status of LGGs and their sensitivity to immunotherapy.

In this study, by analyzing the data of the Cancer Genome Atlas (TCGA) and the Chinese Glioma Genome Atlas (CGGA), we found tenascin-C (TNC) was associated with the prognosis of LGG patients and could reflect the status of the tumor immune microenvironment. We then examined the correlation between TNC and LGGs tumor immune microenvironment in tumor specimens and patients’ serum, and evaluated TNC as a predictor of the immunotherapeutic efficacy in LGG patients.

## Methods

### Patients

We enrolled 62 glioma patients (grade II and grade III) receiving operation at Tongji Hospital of Huazhong University of Science and Technology (Wuhan, China) from January 2016 to July 2021. Brain tissues from 6 normal subjects were used as controls. The clinical data were detailed in **Table 1** and **Supplementary Tables 1, 2**.

**Table 1:** Information of participants in this trial.

Clinical characteristics of patients subjected to IHC testing	
Characteristic	N=30
Grade	
WHO II	16
WHO III	14
Histology	
Astrocytoma	28
Oligodendroglioma	2
Gender	
Male	11
Female	19
Age (years)	
Median	44
Range	13-63
Location	
Frontal lobe	13
Temporal lobe	5
Parietal lobe	2
Multiple lobes	10
Overall survival (days)	
Median	637.5
Range	44-1091
Clinical characteristics of other participants.	
Characteristic	N=38
Grade	
Normal brain tissues	6
WHO II	15
WHO III	17
Histology	
Astrocytoma	29
Oligodendroglioma	3
Gender	
Male	22
Female	16
Age (years)	
Median	48
Range	15-73
Location	
Frontal lobe	14
Temporal lobe	13
Lateral ventricle	2
Multiple lobes	9

### Datasets and Bioinformatics Tools and Algorithms

RNA-seq data of 530 LGG samples and their clinicopathological information were down-loaded from the TCGA database (https://portal.gdc.cancer.gov/projects/TCGA-LGG). RNA-seq data of 441 LGG samples and the survival information were downloaded from the CGGA database (http://www.cgga.org.cn/download.jsp) (**Supplementary Table 3**).

We used the following modules of R packages (1): GSVA (2), Limma (RRID : SCR_010943) (3), GSEABase package, to conduct ssGSEA analysis. Based on the scores obtained, the hierarchical cluster method was utilized for clustering, and a ggplot package was employed for drawing heatmaps.

The lists of immune-related genes were downloaded from the ImmPort (https://www.immport.org/shared/home), which is the basis of ssGSEA algorithms. The scores of co-gene sets yielded from the scale could distinguish the status of immune microenviroment of tumors. Moreover, for verification, Estimation of STromal and Immune cells in MAlignant Tumour tissues using Expression data (ESTIMATE), an algorithm that analyzes the overall tumor microenvironment, was employed. By screening out two signatures, i.e., stromal signature and immune signature, we could calculate the scores of stromal and immune cells. In the end, an ESTIMATE score was obtained and was used for the analysis of tumor purity. Higher ESTIMATE scores, immune scores, and stromal scores reflect the higher content of each component in the tumor microenvironment, and the lower tumor purity.

In addition, for the evaluation of infiltration of immune cells in the microenvrionment of tumors, given that the abundance of immune cells and the consistency of data analyses, we used Immune Cell Abundance Identifier (ImmuCellAI), an ssGSEA-based online analysis tool, to calculate infiltration levels of 24 immune cells by uploading transcriptome data (http://bioinfo.life.hust.edu.cn/ImmuCellAI#!/) (13). Since ImmuCellAI has no well-established classification of glioma-associated macrophages (GAMs), and GAM plays a pivotal role in glioma microenviroment, we also employed the XCELL methodology contained in TIMER(http://timer.comp-genomics.org/) to assess the level GAM infiltration and its impact on the prognosis of glioma, with an attempt to add data to ImmuCellAI.

Tumor Immune Dysfunction and Exclusion (TIDE) is a method that is designed to predict the immune escape of tumor and efficacy of immuno-therapies, and a higher tumor TIDE score is predictive of less favorable prognosis of immuno-therapies (14). By assessing two characteristic gene signatures (i.e., T cell dysfunction and T cell exclusion), the TIDE scores can be obtained (14). TIDE scores were evaluated online (https://tide.dfci.harvard.edu/), by using Z-transformed CGGA and TCGA transcriptome data.

For the overall expression profile data of CGGA and TCGA, we downloaded the software GSEA_Win_4.1.0 to obtain the results (http://www.gsea-msigdb.org/gsea/downloads.jsp), and the genes with p-value < 0.05 and |log_2_FC| > 1) were identified by running the limma package of R, and immune-related differentially-expressed genes were assessed by applying Gene Ontology (GO) and Kyoto Encyclopedia of Genes and Genomes (KEGG, RRID: SCR_012773) analyses with the clusterProfiler (RRID : SCR_016884) package of R.

### Identification of Target Genes

In this study, we first performed a single sample gene set enrichment analysis (ssGSEA) on the transcriptome data of TCGA and CGGA. Then the subjects were clustered on the basis of the ssGSEA scores. In terms of the scores of the LGG immune microenvironment, the LGG population was divided into a high-immunity subgroup and a low-immunity subgroup. Subsequently, we found 7 genes to identify the patients with high-immunity infiltration, and they met all the following screening criteria: They were (i) highly expressed in LGGs, (ii) over-expressed in the immunity-high subgroup, (iii) readily available for clinical diagnosis; and (iv) subjects with preferential expression of the genes had a poor prognosis. So, we finally identified our target, *i.e*., tenascin-C (TNC), on the basis of the relative expression of these 7 genes (LGGs *vs.* normal brain tissues).

### Antibodies

Antibodies used in immunohistochemistry: Anti-CD68 (1:1000, Sigma, Cat#HPA048982, RRID : AB_2680587), anti-CD4 (1:500, Abcam, Cat#ab133616, RRID : AB_2750883), anti-CD8 (1:200, Cell Signaling Technology, Cat#85336,RRID : AB_2800052), anti-FOXP3 (1:100, Abcam, Cat#ab20034, RRID : AB_445284), anti-CD206 (1μg/ml, Abcam, Cat#ab64693, RRID : AB_1523910), anti-TNC (1:100, Abcam, Cat#ab108930, RRID : AB_10865908).

Antibodies used in immunofluorescence: Anti-TNC (1:50, Santa Cruz Biotechnology, Cat#SC13578, RRID : AB_628341), anti-CA9 (1:100, NOVUS, Cat#NB100-417, RRID : AB_10003398), anti-Iba1 (1:100, Cell Signaling Technology, Cat#17198S, RRID : AB_2820254). Secondary Antibodies: Anti-mouse IgG (1:100, Invitrogen, Cat#2266877), anti-rabbit IgG (1:200, Invitrogen, Cat#2273718).

Antibodies used in western blotting: Anti-TNC (1:500, Santa Cruz Biotechnology, Cat#B1120),anti-CD11b (1:1000, Cell Signaling Technology, Cat#17800S), anti-p-STAT3 (1:1000, Cell Signaling Technology, Cat#9145S, RRID : AB_2491009), anti-α-Tubulin (1:5000, ABclonal, Cat#AC012, RRID : AB_2768341). Secondary Antibodies: Anti-rabbit IgG (1:1000, Cell Signaling Technology, Cat#7074P2, RRID : AB_2099233), anti-mouse IgG (1:1000, Cell Signaling Technology, Cat#7076P2, RRID:AB_330924).

### Immunohistochemistry

ABC kit was reportedly used for IHC staining of tissue sections and DAB detection (15). The histochemical scoring (H-SCORE) was employed to measure the expression level of a target protein.

### Immunofluorescence Staining

Tissues were stained with IF method as previously described (15). Briefly, 4% paraformaldehyde was used for the fixation of tumor specimens for 15 min. The samples were blocked with 10% donkey serum containing 0.5% Triton X-100 (Bio-Frox) at room temperature for 1 h. The samples were then incubated overnight at 4°C with the primary antibody. Then they were incubated for 1 h at room temperature with the corresponding secondary antibody. DAPI was utilized for nuclear staining. Images were acquired by using a fluorescence microscope (Olympus). IF staining was performed in triplicate in LGG specimens.

### Immunoblotting

Immunoblotting was conducted as reported previously (15). Briefly, glioma tissues were ground and lysed by using RIPA buffer supplemented with protease and phosphatase inhibitors. Protein samples were separated by SDS-PAGE and transferred onto PVDF membranes. Proteins were labeled with primary antibodies overnight at 4°C, and then the secondary antibodies. ECL kit was used to visualize the immunoreactivity.

### Quantitative Real-Time PCR

Total RNA was isolated from glioma tissues with TRIZOL (Invitrogen). According to the manufacturer’s instructions, cDNA was synthesized by using HIScript^®^ II Q RT SuperMix (#R233-01, Vazyme). qRT-PCR was performed by using ChamQ SYBR Master Mix (#Q311-02/03, Vazyme) on the Applied Biosystems StepOnePlusTM Real-Time PCR System. Results were normalized to the β-actin gene and calculated with the method (Total number of cycles/ΔCt). All primer sequences are listed in **Supplementary Table 4**.

### ELISA

Coagulant-free venous blood (2 ml) was taken, coagulated at room temperature for 2 h and centrifuged at 1000 g for 15 min. After centrifugation, serum was collected and the assay was conducted immediately. The concentrations of TNC were measured in duplicate with ELISAs according to the instructions of CUSABIO kit (Catalog Number: CSB-E13125h). The plates were read on a microplate reader at 450 nm.

### Statistical Analysis

All statistical analyses were performed by employing R software package (version 4.0.2) or GraphPad Prism (version 7.0, RRID : SCR_002798), and all summary data were presented as mean± standard error. For the data with normal distribution, we used the unpaired Student’ *t* test for comparison between two groups and one-way ANOVA for multiple group comparison. For non-normally distributed data, Wilcoxon rank-sum test was utilized to make the comparison between two groups and the K-W test was used to compare multiple groups of independent samples. Moreover, the Log-rank test was employed to generate and compare Kaplan-Meier curves. *p*<0.05 was considered to be statistically significant.

## Result

### Screening of Related Genes for Predicting Immune Microenvironment Status in LGGs

**Figure 1:**
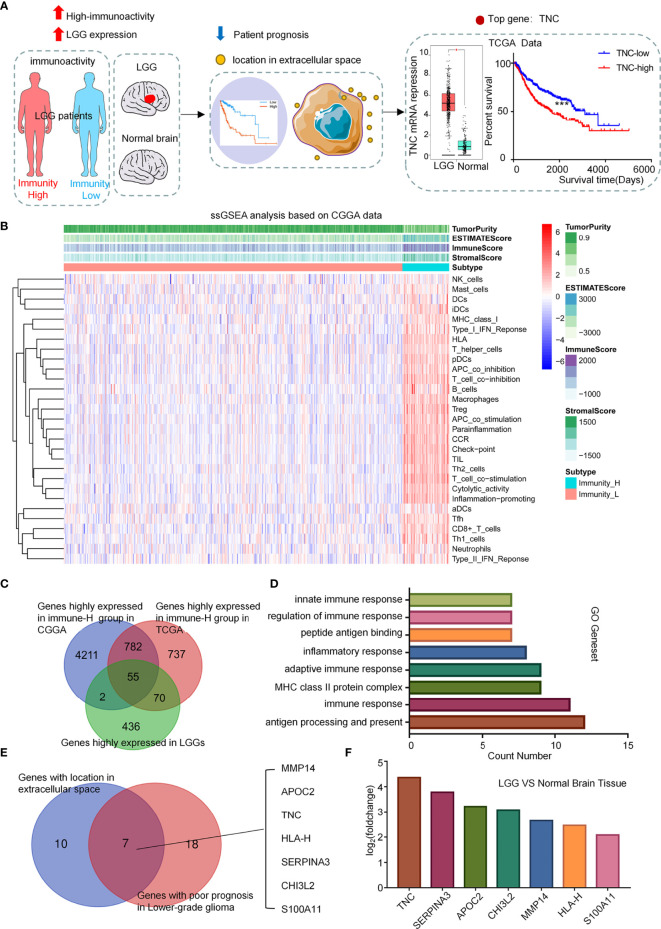
Screening of related genes for predicting immune microenvironment in LGGs. **(A)** Diagram showing the bioinformatical strategies to identify candidates that can reflect the status of LGG immune microenvironment and predict the prognosis. **(B)** Clustering heatmap based on ssGSEA scores of 29 immune-related gene sets in CGGA database. **(C)** Venn diagram showing genes that were highly expressed in both the immunity-high subgroup and LGG patients. **(D)** Functional gene-set enrichment analysis of 55 genes selected in **(C)**. **(E)** Venn diagram showing the intersection between the genes located in extracellular space and the genes related to poor prognosis in LGGs as calculated by the Log-rank test. **(F)** Immune-related target genes ranked in terms of relative gene expression level (LGGs *vs*. normal brain tissues).

In order to know the status of the LGG tumor immune microenvironment more conveniently, we analyzed the CGGA and TCGA database and obtain the relevant genes in terms of their clinical implication and the nature of the encoded proteins (**Figure 1A**). We performed ssGSEA analysis on 29 immunity-related gene sets, and then sub-grouped the LGG patients into low-immunity subjects and high-immunity ones in terms of ssGSEA scores (**Figure 1B** and **Supplementary Figure 1A**). In order to verify the reliability of the grouping, we used the ESTIMATE algorithm to evaluate the immune microenvironment of LGG. It was found that the ESTIMATE scores, stromal scores, and immune scores of the high-immunity subgroup were higher, and the tumor purity was lower (*p*<0.001) (**Supplementary Figures 1B, C**). We found that the prognosis of the high-immunity subgroup was unfavorable, even in patients who had undergone radiotherapy or chemotherapy (*p*<0.05) (**Supplementary Figures 2A, B**). We also found that the proportion of patients with IDH mutations was greater in the low-immunity subgroup (**Supplementary Figure 2C**), and the patients with IDH mutations had a worse prognosis when immune infiltration was higher (**Supplementary Figure 2D**). Then, we selected genes with (1) high expression in the LGG high immune subgroup, and (2) elevated expression in LGG tissue *versu*s normal brain tissue. In the end, we identified 55 genes (**Figure 1C**). We performed GSEA on these genes, and the results showed that they were enriched in immunity-related pathways (**Figure 1D**). Then, we found 7 genes of interest from these genes encoded for protein secretion that lead to poor prognosis (**Figure 1E**). Finally, we found that TNC had the highest content in tumors and, therefore, used it as the research target (**Figure 1F**).

### TNC Was Associated With LGGs Immune Microenvironment

**Figure 2:**
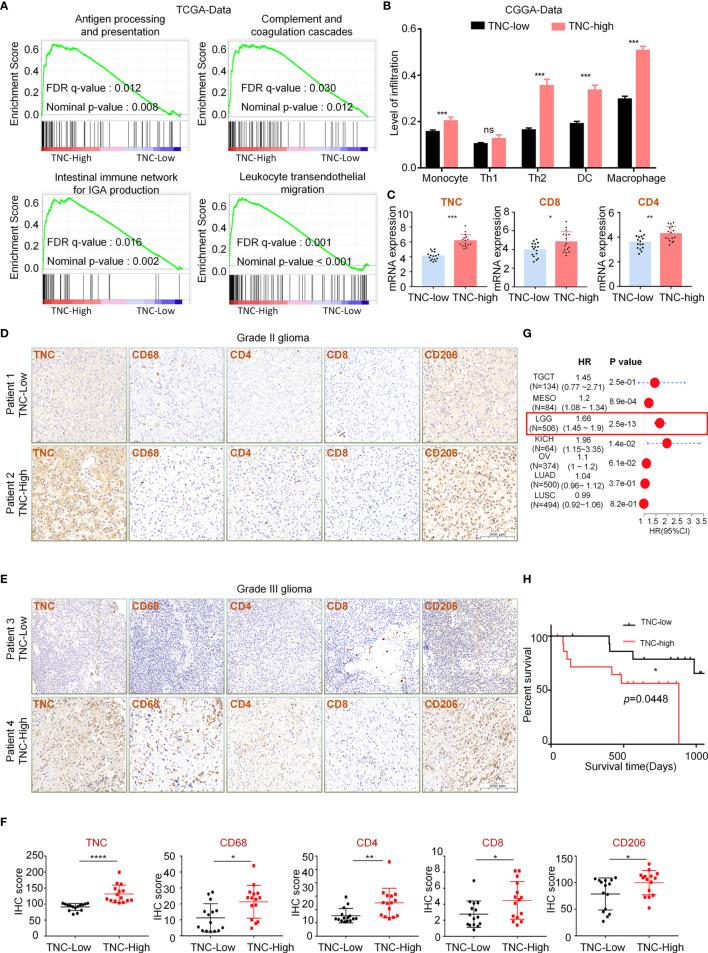
TNC was associated with LGGs immune microenvironment **(A)** Functional gene-set enrichment analysis of TNC used TCGA transcriptome data. **(B)** Histogram describing the level of immune cell infiltration as calculated by using the ImmuCellAI theory in different TNC expression groups. (*** *p* < 0.001, ns, not significant) **(C)** qRT-PCR analysis of mRNA expression of CD4 and CD8 and TNC in 32 LGG specimens grouped by TNC mRNA level. (* *p* < 0.05; ** *p* < 0.01; *** *p* < 0.001) **(D, E)** IHC staining of TNC, tumor-associated macrophages markers (CD68 and CD206), and T cell markers (CD4 and CD8) in grade II **(D)** and grade III **(E)** LGG specimens. Representative images are shown. (Scale bars = 200 *um*) **(F)** Scatter plot showing the distribution of CD68, CD206, CD4 and CD8 IHC scores (H-SCORE) in the two subgroups grouped based on the median of the TNC staining score. (* *p* < 0.05; ** *p* < 0.01; ***p < 0.001, ****p < 0.0001, *n* = 30) **(G)** The forest plot showing the single-factor logistic regression analysis of the prognosis of TNC in TCGA pan-cancer data. (LGG: HR=1.66) **(H)** Kaplan-Meier curve depicting the clinical value of TNC in 30 LGG patients with follow-up information. (* *p* < 0.05).

To explore the influence of TNC on the immune microenvironment status of LGGs, we conducted GSEA in the TCGA database. The results exhibited that immunity-related pathways were predominantly enriched in the high-TNC subgroup (*p*<0.05) (**Figure 2A**). We found that the high-TNC subgroup had higher ESTIMATE scores, stromal scores, immune scores, and lower tumor purity (*p*<0.001) (**Supplementary Figure 3A**). Immune cell infiltration also was increased in the high-TNC subgroup (**Figure 2B**). To verify the aforementioned findings, we conducted qRT-PCR on 32 fresh tumor tissues and revealed that mRNA expression of CD4 and CD8 was higher in the high-TNC subgroup (*p*<0.05) (**Figure 2C**). IHC showed that the expression of T cell-related molecules (CD4, CD8) and macrophage-related molecules (CD68, CD206) was up-regulated in the high-TNC subgroup (*p*<0.05) (**Figures 2D** **–F** and **Supplementary Figures 3B, C**). We found that in LGG, when the infiltration of CD4^+^ and CD8^+^ cells increased, the prognosis of the patient was poor (*p*<0.05) (**Supplementary Figure 3D**). As to the implication of TNC in the prognosis of LGG patients, we found that the Hazard Ratio (HR) of TNC in LGG was significantly higher (HR=1.66, *p*<0.05) (**Figure 2G**). Then, we followed up 30 patients who had been subjected to IHC for survival and found that the survival in the high-TNC subgroup was poor (*p*=0.0448) (**Figure 2H**).

### TNC Was Related to Glioma-Associated Macrophages Infiltration in LGGs

**Figure 3:**
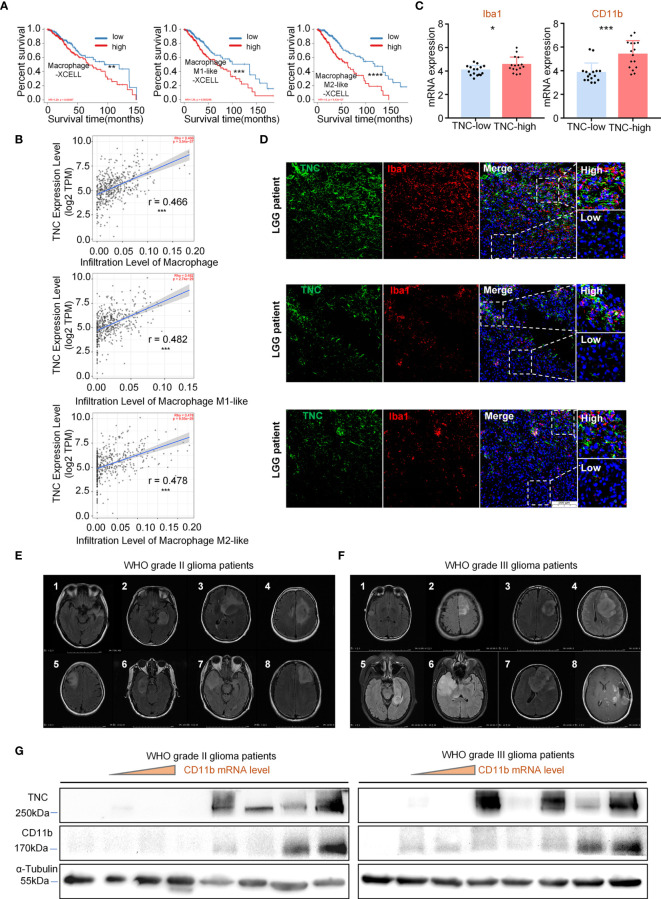
TNC was related to GAM cell infiltration in LGGs **(A)** Kaplan-Meier survival analysis showing that the infiltration level of macrophages, M1-like cells, and M2-like cells with the prognosis of LGG patients in the TIMER database using the XCELL method. (** *p* < 0.01; *** *p* < 0.001; **** *p* < 0.0001) **(B)** The scatter plot indicating the correlation between TNC expression level (log_2_TPM) and the infiltration level of macrophages, M1-like cells, and M2-like cells in LGG patients using the TIMER database. (*** *p*<0.001) **(C)** qRT-PCR analysis of Iba1 and CD11b mRNA expression in 32 patients grouped by TNC mRNA level. (* *p* < 0.05; *** *p* < 0.001) **(D)** IF staining of TNC (green) and Iba1 (red) in human LGG samples. Representative images are shown. (Scale bars = 200 *um*) **(s)** MRI images of patients with grade II **(E)** and grade III **(F)** gliomas.(*n*=16) **(G)** Immunoblotting of TNC and CD11b in grade II and III glioma patients sorted by CD11b mRNA level. (The samples correspond to the patients in **(E, F)**.

To explore the infiltration of different GAM phenotypes had any impact on the survival of LGG patients, we performed a survival analysis in the patients and the results showed that the prognosis of patients was poor when the total GAM cells, M1-like and M2-like phenotype of GAMs were elevated (*p*<0.05) (**Figure 3A**). However, upon analysis of GBM data, we found that the patients’ prognosis was related to the GAM cell types (**Supplementary Figure 4**). Next, we found that TNC was positively correlated with total GAMs, M1-like and M2-like phenotypes (**Figure 3B**). We used fresh tumor samples for mRNA detection and found that the mRNA expression of GAM-related genes CD11b and Iba1 were increased in the high-TNC subgroup (*p*<0.05) (**Figure 3C**). IF staining indicated that Iba1^+^ cell infiltration bore a correlation with high-TNC expression (**Figure 3D**). Western blotting detection of fresh samples demonstrated that the expression levels of CD11b and TNC protein were comparable (**Figures 3E** **–G**).

### TNC Was Mainly Related to the Immunosuppressive Microenvironment of LGGs

**Figure 4:**
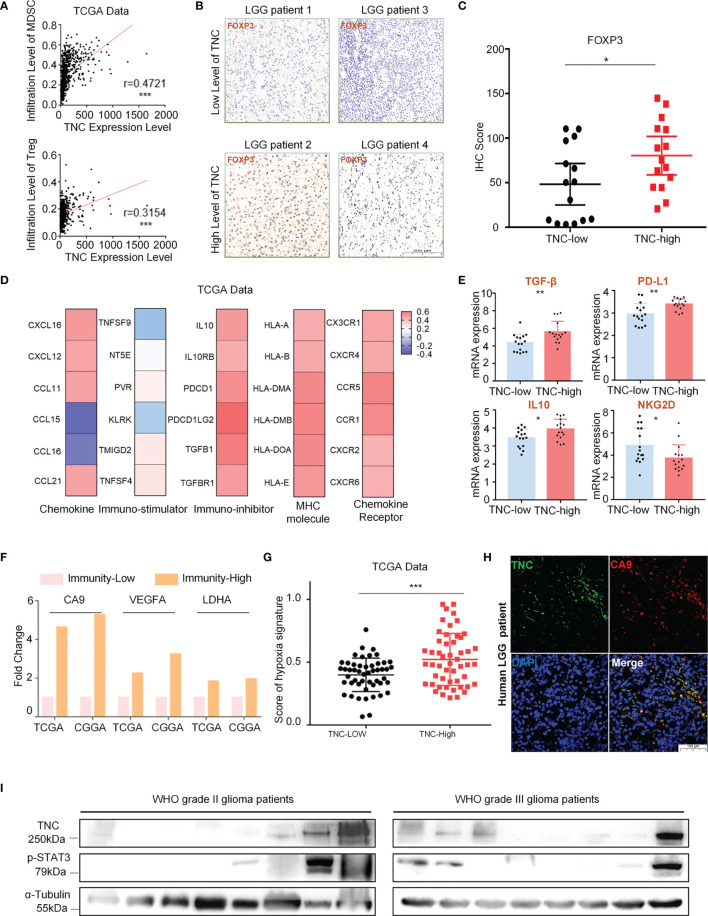
TNC was mainly related to the immunosuppressive microenvironment of LGGs. **(A)** Scatter plot displaying the correlation between TNC mRNA level and MDSC/Treg cell level. (*** *p* < 0.001) **(B)** IHC staining of FOXP3 protein in LGG specimens in terms of TNC IHC scores level. (Scale bars = 200 *um*. The sample is consistent with Fig. 2d, 2e) **(C)** Scatter plot showing the distribution of FOXP3 IHC scores in the two subgroups grouped by the median of the TNC IHC score. (*p<0.05) **(D)** Heatmap reflecting the correlation between TNC and chemokine, immuno-stimulator, immune-inhibitor, MHC molecule, and chemokine receptor. **(E)** qRT-PCR analysis of mRNA expression of IL10, TGF-β, PD-L1 and NKG2D in 32 LGG patients grouped by TNC mRNA level. (*p < 0.05; **p < 0.01). **(F)** The histogram showing the mRNA expression difference of hypoxia markers (CA9, VEGFA, and LDHA) in high-immunity subgroup and low-immunity subgroup. **(G)** Scatter plot exhibiting the distribution of hypoxia signature scores in the two subgroups based on the median of TNC mRNA level in TCGA data. (*** *p* < 0.001). **(H)** IF staining of TNC (green) and hypoxia marker (CA9; red) in human LGGs. (Scale bars=100 *um*, representative images are shown) **(I)** Immunoblotting of TNC and p-STAT3 in grade II and III glioma patients. (*n*=16).

Our data exhibited that TNC was positively correlated with myeloid-derived suppressor cells (MDSCs) and regulatory T cells (Tregs) infiltration (**Figure 4A**). IHC showed that the expression of Tregs related protein FOXP3 was elevated when the TNC was highly expressed in LGGs (*p*=0.0393) (**Figures 4B** **, C**). We also found that TNC was positively associated with immunosuppressive factors, immune checkpoints, immune checkpoint receptors, and MHC molecules, such as IL10, TGF-β (TGFB1), PD1 (PDCD1), HLA-DOA, CCL21, among others. However, TNC was negatively correlated with immuno-stimulatory factors TNFSF9 and NKG2D (KLRK) (**Figure 4D** and **Supplementary Figure 5A**). In the low-TNC subgroup, the infiltration of natural killer T cell (NKT), γδT cell, B cell, and other immune effector cells were increased (*p*<0.05) (**Supplementary Figure 5B**). We performed qRT-PCR on fresh LGG samples and found that, in the high-TNC subgroup, the expression of IL-10, TGF-β, PD-L1 was up-regulated, and the expression of immunostimulatory factor NKG2D was down-regulated (*p*<0.05) (**Figure 4E**).

The tumor hypoxic microenvironment is generally believed to be related to immunosuppression (16, 17). We evaluated LGG patients from the TCGA and the CGGA databases and found that the expression of hypoxia-related factors carbonic anhydrase 9 (CA9), vascular endothelial growth factor A (VEGFA), and lactate dehydrogenase A (LDHA) was increased in the high-immunity subgroup (**Figure 4F**). Similarly, we also observed that the subgroup with high-TNC in LGGs scored higher in terms of hypoxia-related signals (**Figure 4G**). We used fresh LGG tumor specimens for IF detection and found that TNC and CA9 were co-expressed (**Figure 4H**). Immunosuppression in glioma is considered to be related to the activation of STAT3 (18–20). We performed Western blot analysis and found that the expression of p-STAT3 was increased when TNC was elevated (**Figure 4I**).

### Serum TNC Could Reflect the Immune Microenvironment of LGGs and Predict the Effect of Immunotherapy.

**Figure 5:**
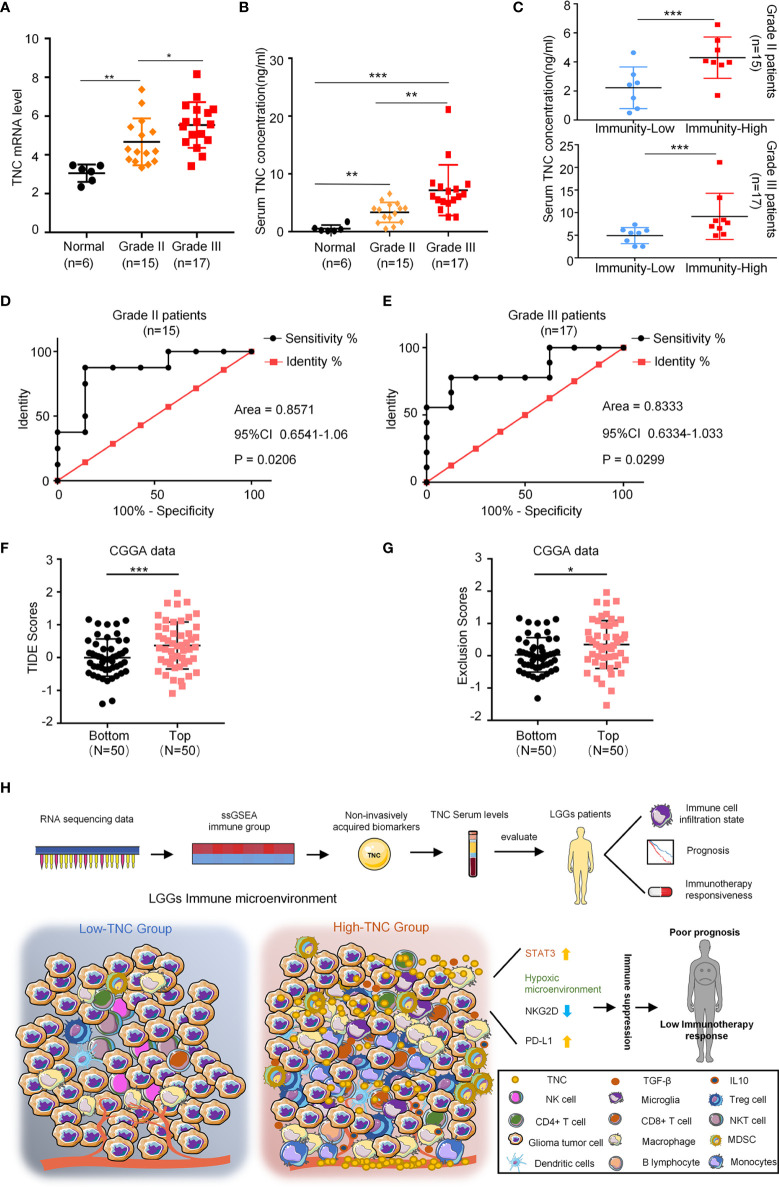
Serum TNC can reflect the immune microenvironment of LGGs and predict the effect of immunotherapy. **(A)** qRT-PCR analysis of mRNA expression of TNC in normal brain tissues (*n* = 6), grade II (*n* = 15) and grade III (*n* = 17) glioma patients. (* *p* < 0.05; ** *p* < 0.01) **(B)** Scatter plot displaying the serum concentration of TNC in normal brain tissues (*n* = 6), grade II (*n* = 15) and grade III (*n* = 17) glioma patients. (** *p* < 0.01; *** *p* < 0.001) **(C)** Scatter plot demonstrating the correction between TNC serum concentration and immunity-status in grade II and III glioma patients (Grouped based on the median of the tumor CD11b mRNA level. ***p < 0.001; *n* = 32) **(D, E)** ROC curves showing the diagnostic value of serum TNC level for distinguishing the status of LGG immune microenvironment. **(F, G)** TIDE scores and T cell exclusion scores in different TNC mRNA expression subgroups. The score between the two subgroups were compared through the Wilcoxon test. (Top: TNC high expression; Bottom: TNC low expression. * *p* < 0.05, *** *p* < 0.001) **(H)** The potential application of TNC in the early diagnosis and individualized treatment of LGG patients.

Our experiments showed that TNC was increased in tumor tissues and blood of LGG patients (*p*<0.05) (**Figures 5A** **, B**). We divided LGG patients subjected to ELISA into low-immunity subgroups and high-immunity subgroups in terms of CD11b mRNA in tumors. In grade II and III gliomas, we found serum TNC level was increased in the high immune subgroup (*p*<0.05) (**Figure 5C**). ROC analysis found that serum TNC content could be used to assess the status of tumor immune microenvironment in patients with grade II (AUC=0.8571; 95% CI: 0.6541-1.06) and grade III (AUC=0.8333; 95% CI: 0.6334-1.033) gliomas (**Figures 5D** **, E**). Finally, we used the TIDE tool to analyze the CGGA-LGG transcriptome data and found that patients with higher TNC expression registered higher TIDE scores and Exclusion Scores (p<0.05) (**Figures 5F** **, G**). Similarly, TCGA-LGG transcriptome data revealed that higher TNC expression was associated with higher TIDE scores and Dysfunction Scores (p<0.05) (**Supplementary Figure 6**).

## Discussion

Despite advances in the treatment, the overall survival of glioma patients has not been significantly improved in the past few decades (11). Recently research effort has been directed at understanding the interaction between the immunity and the progression of glioma. Moreover, clinicians tried to use immunotherapy to improve the prognosis of glioma. Immunotherapy takes advantages of the specificity and killing mechanism of the immune system to target and remove tumor cells. Nonetheless, in glioma, the key components of the immune microenvironment have experienced substantial change, which leads to tumor immune escape (21, 22). Moreover, the drug resistance of glioma also bears an intimate correlation with the immunosuppressive microenvironment (23, 24). It is of great importance to find a reliable bio-indicator that can mirror the tumor immune microenvironment status and allow for personalized treatment.

In this study, we identified a bio-indicator, *i.e*., TNC, whose expression is associated with chronic inflammation and development of multiple malignancies, including gliomas. TNC regulates cellular adhesion, migration, proliferation and angiogenesis in tumors by binding to its corresponding receptors (25). Previous studies have demonstrated that TNC is implicated in immune regulation and other important biological processes (26). Nevertheless, the effect of TNC on the LGG immune microenvironment remains poorly understood. In this study, we explored the impact of TNC on the immune microenvironment of LGGs.

Previous studies found that infiltrating immune cells in gliomas involve central nervous system (CNS) resident cells (microglia), peripheral macrophages, granulocytes, myeloid-derived suppressor cells, and T lymphocytes, among others. The extensive microglial and macrophagal infiltration in gliomas is collectively referred to as GAM (27). CD11b antigen has been used as a common marker of microglia in most human tissue studies. But macrophages and MDSCs also express CD11b (27). CD68, and Iba-1 are commonly used for the identification of GAMs (27). In this study, we showed that, in LGG, CD11b, CD68, and Iba-1 were also increased in high-TNC subgroup, suggesting that TNC expression was associated with GAM infiltration. In GBM, the GAM functions varied with different polarization phenotypes. M1-like macrophages assault tumor cells *via* phagocytosis and the production of pro-inflammatory cytokines. However, M2-like macrophages are involved in immunosuppression and tumorigenesis (28). M2-like macrophages also increase the release of anti-inflammatory and immunosuppressive cytokines, such as IL10 and TGF-β (27, 28). However, our findings suggested that the different polarization phenotypes of GAMs in LGGs exerted no influence on the prognosis of patients. TNC is believed to be related to the polarization of GAMs in GBM (25). The expression of TNC in LGGs was found to be associated with the infiltration of GAMs, no matter M1 or M2. These results indicated that the different types of GAM in LGGs might not have much influence on GAM function, and TNC might elicit GAM infiltration, but did not affect cell polarization.

MDSCs, a heterogeneous population of immature myeloid cells, participate in the development of tumor-induced immunosuppression (29). For instance, MDSCs can release immunosuppressive cytokines and inhibit the activity of T cells by activating STAT3, thereby affecting immunosuppression (18–21). We showed that the infiltration of MDSCs was increased in the high-TNC subgroup. Moreover, STAT3 activation was also enhanced in the high-TNC subgroup. These results suggested that in LGGs, TNC is released by tumor cells possibly to induce MDSC infiltration and activate STAT3 to produce immunosuppression. The finding and its implications need to be further studied.

In glioma, T cells are functionally impaired and the intensity of T cell infiltration is related to the prognosis of patients (30). We exhibited that in LGGs, the infiltration of CD4^+^ and CD8^+^ cells was increased in the high-TNC subgroup, and, as a consequence, the survival of patients was poor. Treg cells usually account for 5-10% of overall CD4^+^ T cells. Nevertheless, in many cancers, the more the Treg cells, the worse the prognosis (31, 32). This might be ascribed to the fact that Treg cells are able to inhibit the effector T cells. Importantly, Treg cells suppress the T cells by secreting immunosuppressive cytokines and down-regulating costimulatory molecules (33). High-level cytokines, such as TGF-β and IDO, support the sustenance of Tregs in the glioma microenvironment, which facilitate the recruitment and survival of Tregs (34, 35). Surprisingly, the infiltration of Treg cells was increased in the high-TNC subgroup, as TGF-β and IDO did, suggesting that, in LGG, TNC can cause T cell dysfunction and result in immunosuppression.

Although NK cells account for only a small proportion of tumor immune cells, studies have shown that NK cells in the immune microenvironment of glioma were highly cytotoxic to tumor cells (36). Therefore, restoring its anti-tumor cytotoxicity has become the main strategy of immunotherapy for glioma (36). NKG2D is a C-type, lectin-like homodimeric receptor expressed by human NK, γδT, and CD8^+^ αβT cells. The activation of NKG2D can lead to the production of pro-inflammatory cytokines, such as IFN-γ, and the release of cytotoxic particles, thereby killing tumors (37). Our study suggested that the infiltration of NK cells and effector T cells was reduced in the high-TNC subgroup, and so was the expression of NKG2D. Therefore, the expression of TNC in LGGs might inhibit the activity of NK cells and effector T cells, leading to immune escape of tumors.

The viability of tumor cells and the response to therapeutic drugs are multifactorial and the hypoxic microenvironment is one of the important factors. In fact, hypoxia is also a signature of the glioma microenvironment (38). The adaptation of glioma cells to the hypoxic microenvironment is mediated by hypoxia-inducible factors (HIFs). Activation of HIFs can induce the accumulation of immune cells in tumors and is also associated with immunosuppression, resulting in poor prognosis (17, 39). Our results showed that the expression of hypoxia-related factors was increased in the LGG high-immunity subgroup and high-TNC subgroup. Previous studies have shown that a hypoxic microenvironment could regulate the expression of TNC (40). We are led to speculate that in LGG tumors, TNC goes up under hypoxic microenvironment, leading to an immunosuppressive microenvironment.

At present, no serum markers are available that can reflect the immune microenvironment status of LGG tumors. In this study, we found that TNC could be detected in serum and its level was indicative of the immunosuppressive microenvironment of LGGs. More significantly, we successfully predicted that the immunotherapeutic effect on high-TNC patients was poor. This suggests that we can predict the prognosis of LGG patients and guide immunotherapy by detecting the level of serum TNC. In the future, whether TNC can be used as a therapeutic target to improve the efficacy of radiotherapy, chemotherapy, and immunotherapy of LGG, warrants further studies.

In conclusion, TNC is a promising immunity-related prognostic indicator for LGGs. Stratification in terms of TNC helps distinguish patients with different immune status and predict their prognosis.

## Data Availability Statement

The original contributions presented in the study are included in the article/**Supplementary Material**. Further inquiries can be directed to the corresponding authors.

## Ethics Statement

The studies involving human participants were reviewed and approved by All samples were harvested upon obtaining written consent from the subjects in accordance with a protocol approved by the Research Ethics Committee of Tongji Hospital, Tongji Medical College, Huazhong University of Science and Technology, Wuhan, China (Serial no. TJ-IBR20181111). Written informed consent to participate in this study was provided by the participants’ legal guardian/next of kin.

## Author Contributions

DG and XY conceived the study and verified the data. PZ and GL were involved in implementation of the project, preparation of the manuscript and analysis of data. JH conducted the experiments. SC collected and synthesize data. PP and BW revised the manuscript. All authors read and approved the final version.

## Funding

This project was supported by the National Natural Science Foundation of China, NO. 81874086 and NO. 82072797; Supported by Program for HUST Academic Frontier Youth Team; Supported by Huazhong University of Science and Technology Independent Innovation Research Fund Project, NO. 2019kfyXJJS187.

## Conflict of Interest

The authors declare that the research was conducted in the absence of any commercial or financial relationships that could be construed as a potential conflict of interest.

## Publisher’s Note

All claims expressed in this article are solely those of the authors and do not necessarily represent those of their affiliated organizations, or those of the publisher, the editors and the reviewers. Any product that may be evaluated in this article, or claim that may be made by its manufacturer, is not guaranteed or endorsed by the publisher.
