# Supplementary material for: Tenascin-C can Serve as an Indicator for the Immunosuppressive Microenvironment of Diffuse Low-Grade Gliomas
Source: Front Immunol. 2022 Mar 16;13:824586. doi: 10.3389/fimmu.2022.824586 (PMC8966496; doi:10.3389/fimmu.2022.824586)
Supplement: Supplementary file 2 [file Presentation_1.pptx]

## Slide 1
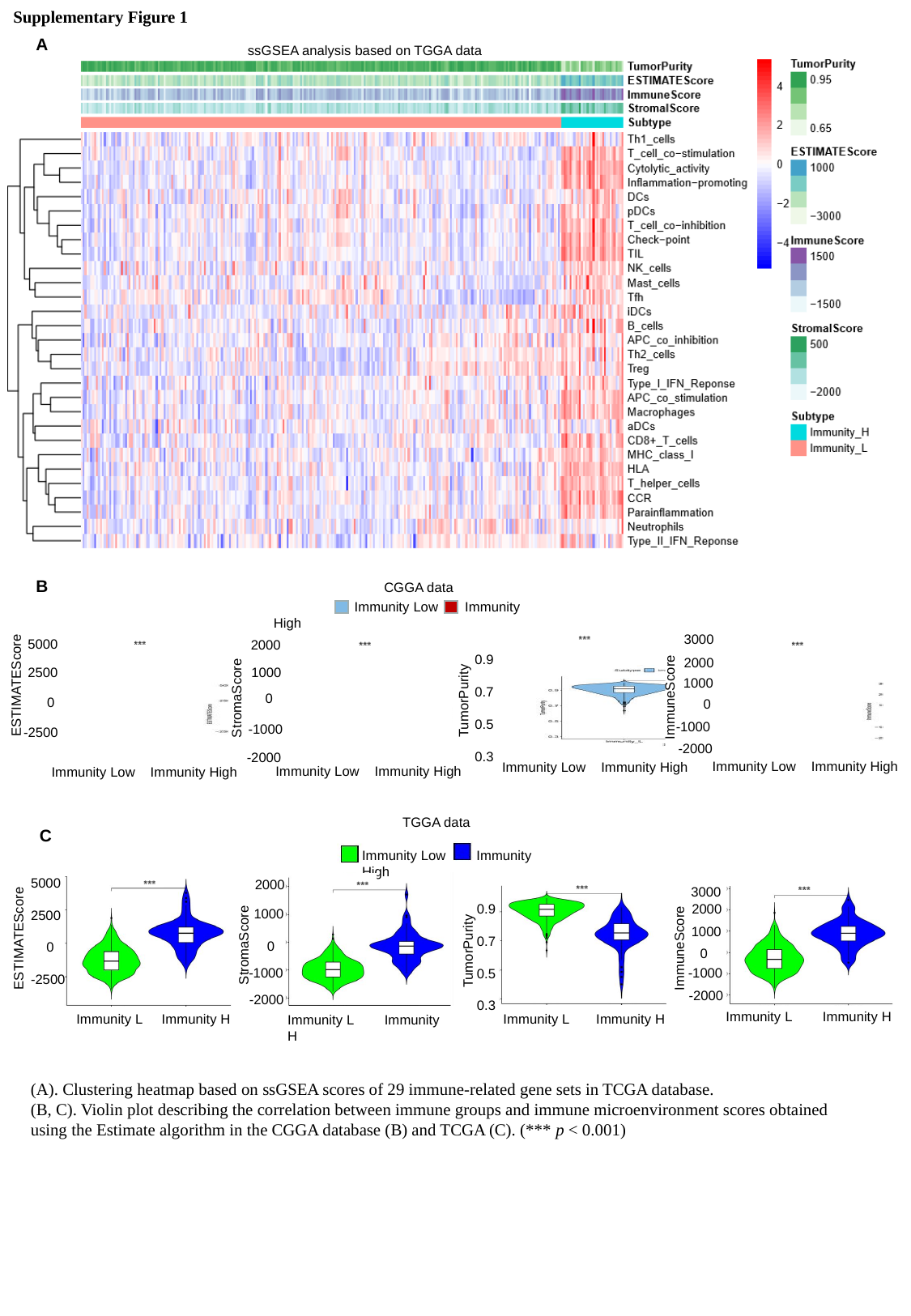

Supplementary Figure 1
A
ssGSEA analysis based on TGGA data
B
CGGA data
 Immunity Low Immunity High
3000
2000
1000
ImmuneScore
0
-1000
-2000
Immunity Low Immunity High
TumorPurity
Immunity Low Immunity High
0.9
0.7
0.5
0.3
5000
2000
1000
StromaScore
0
-1000
-2000
Immunity Low Immunity High
2500
ESTIMATEScore
0
-2500
Immunity Low Immunity High
TGGA data
Immunity Low Immunity High
C
5000
2500
0
-2500
ESTIMATEScore
Immunity L Immunity H
2000
1000
StromaScore
0
-1000
-2000
Immunity L Immunity H
3000
0.9
0.7
0.5
0.3
2000
1000
ImmuneScore
TumorPurity
0
-1000
-2000
Immunity L Immunity H
Immunity L Immunity H
(A). Clustering heatmap based on ssGSEA scores of 29 immune-related gene sets in TCGA database.
(B, C). Violin plot describing the correlation between immune groups and immune microenvironment scores obtained using the Estimate algorithm in the CGGA database (B) and TCGA (C). (*** p < 0.001)

## Slide 2
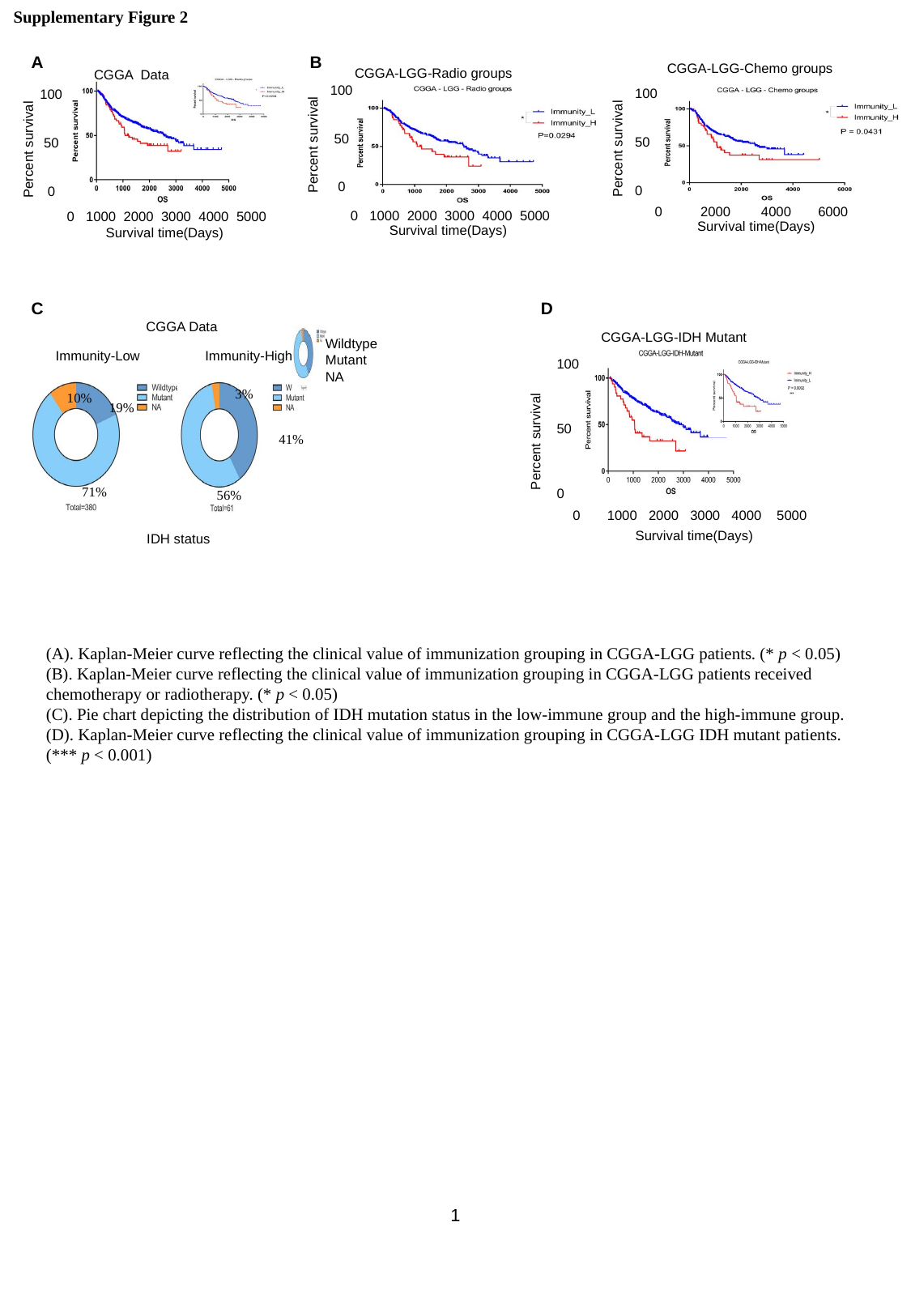

Supplementary Figure 2
A
B
CGGA-LGG-Chemo groups
CGGA Data
CGGA-LGG-Radio groups
100
50
0
Percent survival
100
50
0
100
50
0
Percent survival
Percent survival
0 2000 4000 6000
0 1000 2000 3000 4000 5000
0 1000 2000 3000 4000 5000
Survival time(Days)
Survival time(Days)
Survival time(Days)
C
D
CGGA Data
Wildtype
Mutant
NA
Immunity-Low Immunity-High
IDH status
CGGA-LGG-IDH Mutant
100
50
0
Percent survival
0 1000 2000 3000 4000 5000
Survival time(Days)
3%
10%
19%
41%
71%
56%
(A). Kaplan-Meier curve reflecting the clinical value of immunization grouping in CGGA-LGG patients. (* p < 0.05)
(B). Kaplan-Meier curve reflecting the clinical value of immunization grouping in CGGA-LGG patients received chemotherapy or radiotherapy. (* p < 0.05)
(C). Pie chart depicting the distribution of IDH mutation status in the low-immune group and the high-immune group.
(D). Kaplan-Meier curve reflecting the clinical value of immunization grouping in CGGA-LGG IDH mutant patients. (*** p < 0.001)
1

## Slide 3
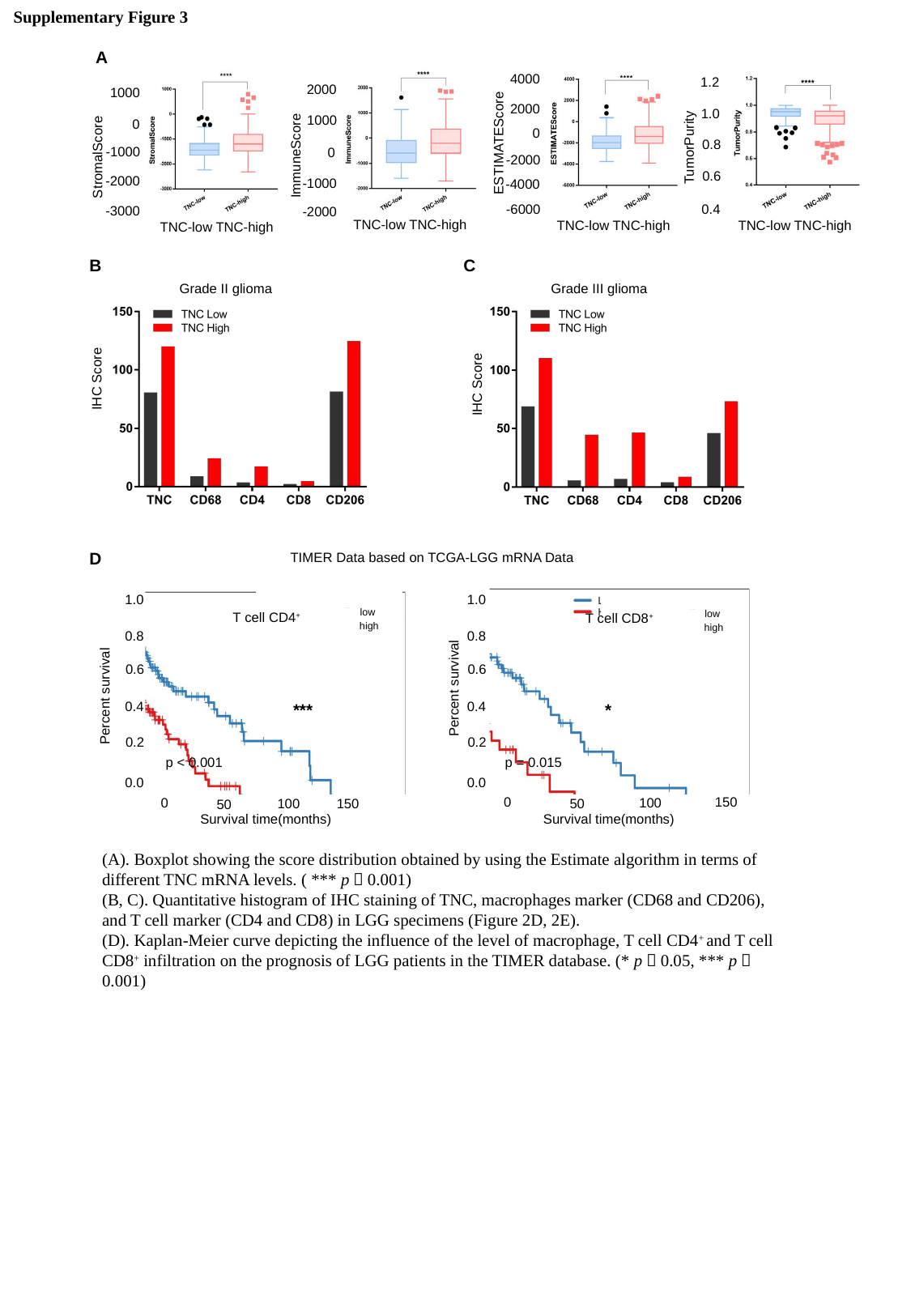

Supplementary Figure 3
A
2000
1000
1000
0
-1000
0
ImmuneScore
StromalScore
-2000
-1000
-3000
-2000
TNC-low TNC-high
TNC-low TNC-high
4000
2000
0
ESTIMATEScore
-2000
-4000
-6000
TNC-low TNC-high
1.2
1.0
0.8
TumorPurity
0.6
0.4
TNC-low TNC-high
B
C
 Grade II glioma
 Grade III glioma
IHC Score
IHC Score
D
TIMER Data based on TCGA-LGG mRNA Data
1.0
0.8
0.6
0.4
0.2
0.0
1.0
0.8
0.6
0.4
0.2
0.0
low
high
low
high
T cell CD4+
T cell CD8+
Percent survival
Percent survival
***
*
p = 0.015
p < 0.001
0
150
100
0
150
100
50
50
Survival time(months)
Survival time(months)
(A). Boxplot showing the score distribution obtained by using the Estimate algorithm in terms of different TNC mRNA levels. ( *** p＜0.001)
(B, C). Quantitative histogram of IHC staining of TNC, macrophages marker (CD68 and CD206), and T cell marker (CD4 and CD8) in LGG specimens (Figure 2D, 2E).
(D). Kaplan-Meier curve depicting the influence of the level of macrophage, T cell CD4+ and T cell CD8+ infiltration on the prognosis of LGG patients in the TIMER database. (* p＜0.05, *** p＜0.001)

## Slide 4
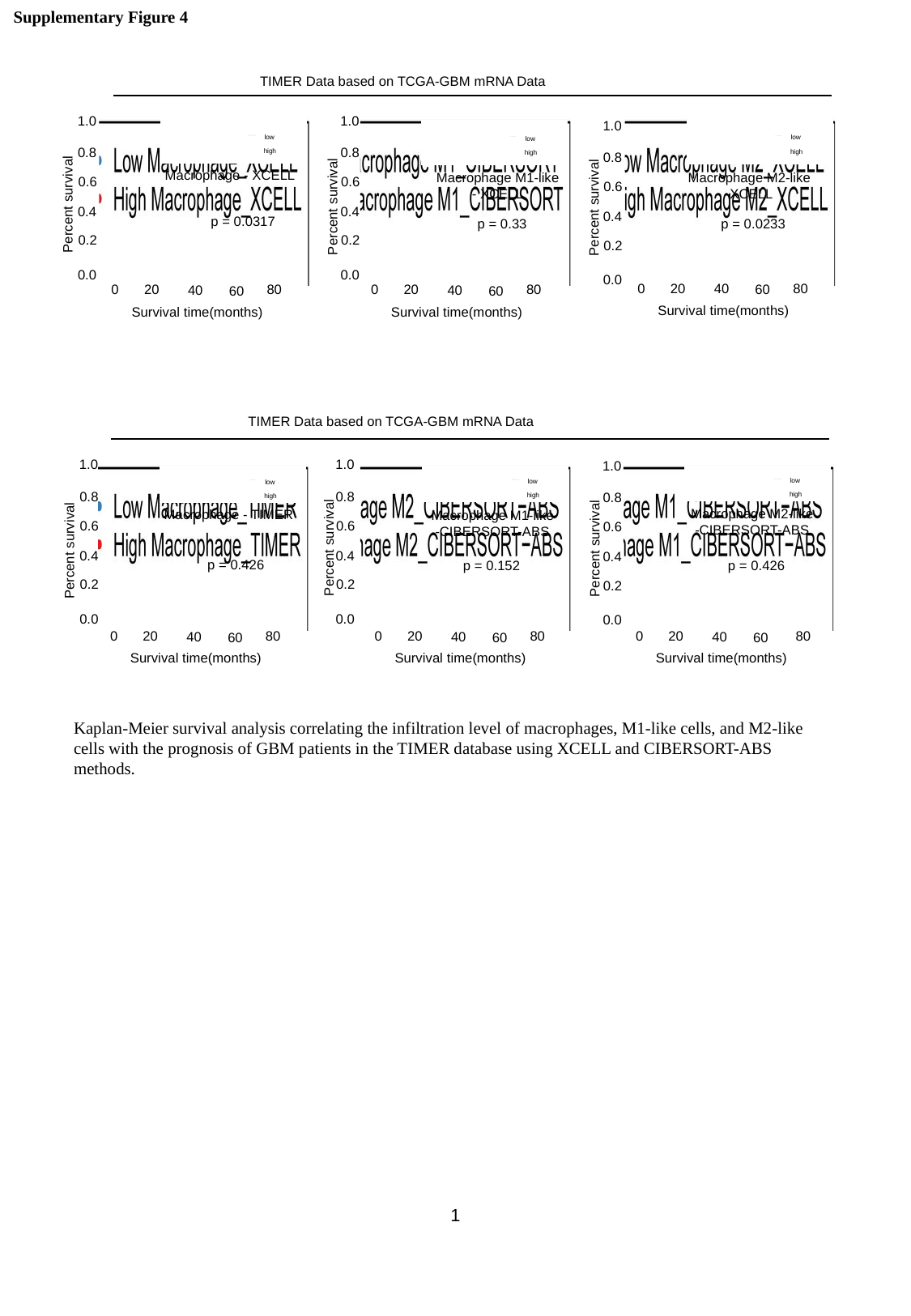

Supplementary Figure 4
TIMER Data based on TCGA-GBM mRNA Data
1.0
0.8
0.6
0.4
0.2
0.0
1.0
0.8
0.6
0.4
0.2
0.0
1.0
0.8
0.6
0.4
0.2
0.0
low
high
low
high
low
high
Macrophage - XCELL
Macrophage M1-like
- XCELL
Macrophage M2-like
-XCELL
Percent survival
Percent survival
Percent survival
p = 0.0317
p = 0.33
p = 0.0233
20
80
0
40
60
Survival time(months)
20
80
0
40
60
Survival time(months)
20
80
0
40
60
Survival time(months)
TIMER Data based on TCGA-GBM mRNA Data
1.0
0.8
0.6
0.4
0.2
0.0
low
high
p = 0.426
20
80
0
40
60
Survival time(months)
1.0
0.8
0.6
0.4
0.2
0.0
1.0
0.8
0.6
0.4
0.2
0.0
low
high
low
high
Macrophage M2-like
-CIBERSORT-ABS
Macrophage - TIMER
Macrophage M1-like
-CIBERSORT-ABS
Percent survival
Percent survival
Percent survival
p = 0.152
p = 0.426
20
80
0
40
60
Survival time(months)
20
80
0
40
60
Survival time(months)
Kaplan-Meier survival analysis correlating the infiltration level of macrophages, M1-like cells, and M2-like cells with the prognosis of GBM patients in the TIMER database using XCELL and CIBERSORT-ABS methods.
1

## Slide 5
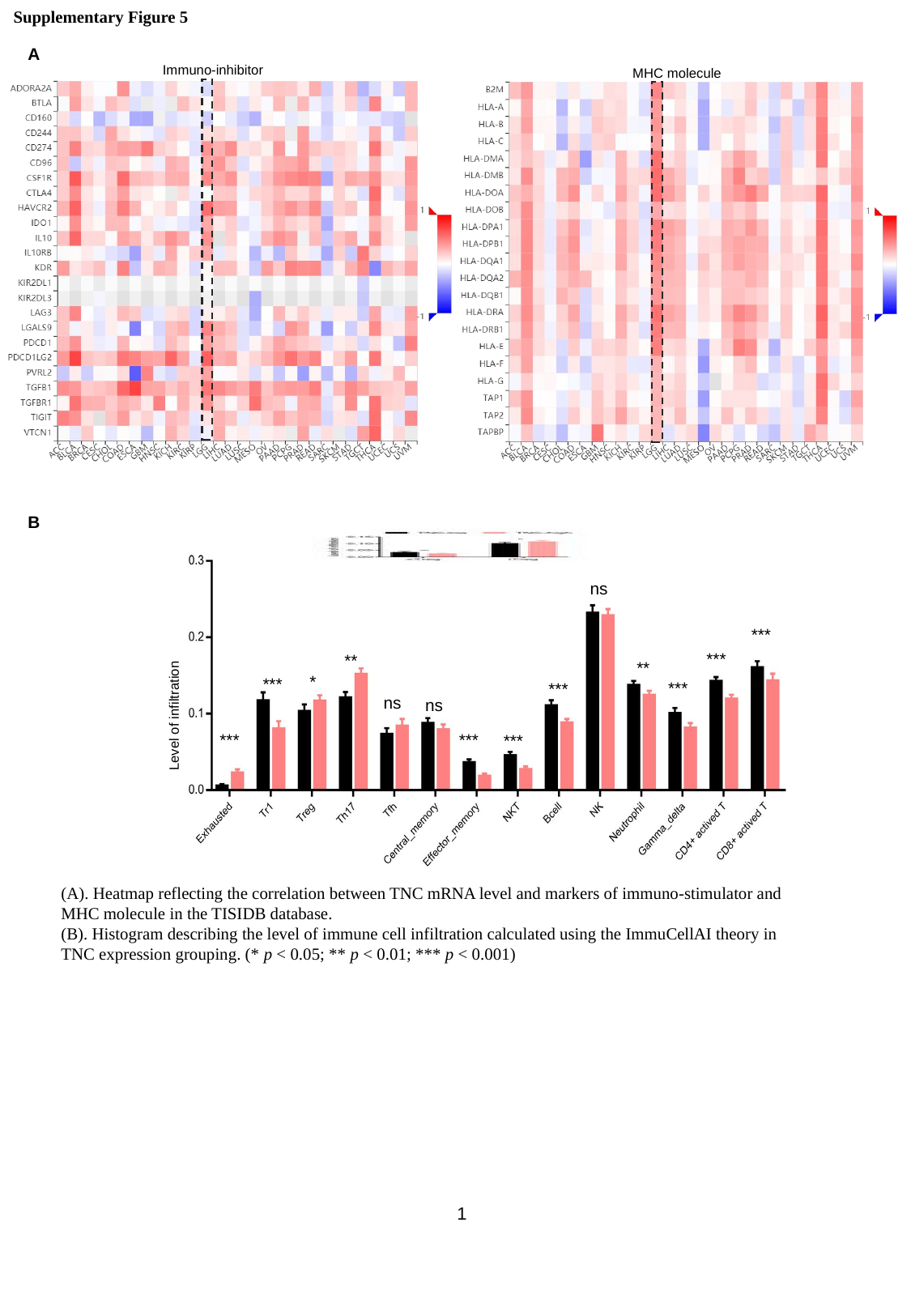

Supplementary Figure 5
A
Immuno-inhibitor
MHC molecule
B
ns
***
***
**
**
Level of infiltration
*
***
***
***
ns
ns
***
***
***
(A). Heatmap reflecting the correlation between TNC mRNA level and markers of immuno-stimulator and MHC molecule in the TISIDB database.
(B). Histogram describing the level of immune cell infiltration calculated using the ImmuCellAI theory in TNC expression grouping. (* p < 0.05; ** p < 0.01; *** p < 0.001)
1

## Slide 6
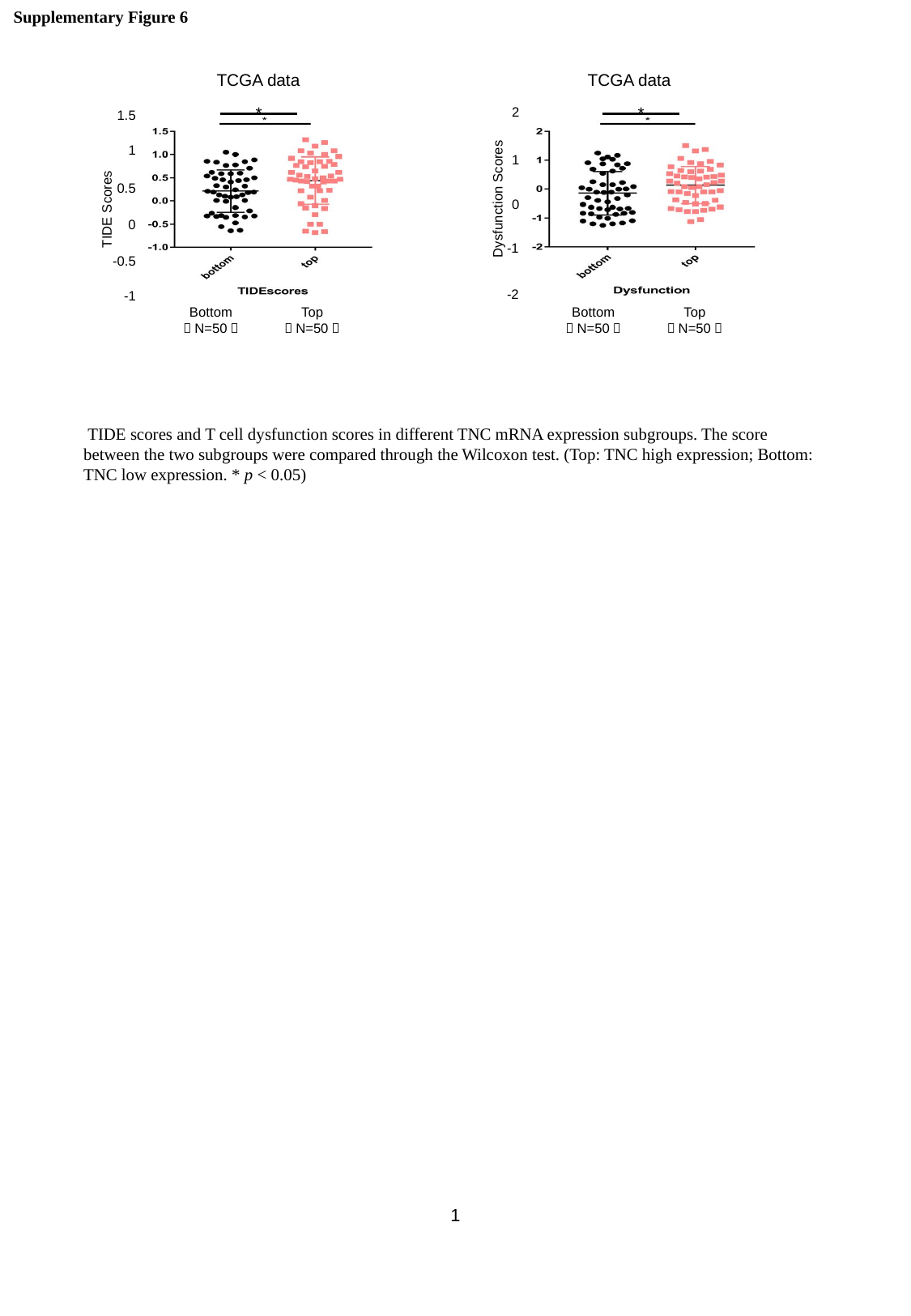

Supplementary Figure 6
TCGA data
TCGA data
*
1.5
1
0.5
0
-0.5
-1
TIDE Scores
Bottom
（N=50）
Top
（N=50）
*
2
1
0
-1
-2
Dysfunction Scores
Bottom
（N=50）
Top
（N=50）
 TIDE scores and T cell dysfunction scores in different TNC mRNA expression subgroups. The score between the two subgroups were compared through the Wilcoxon test. (Top: TNC high expression; Bottom: TNC low expression. * p < 0.05)
1
