# Supplementary material for: Tenascin-C can Serve as an Indicator for the Immunosuppressive Microenvironment of Diffuse Low-Grade Gliomas
Source: Front Immunol. 2022 Mar 16;13:824586. doi: 10.3389/fimmu.2022.824586 (PMC8966496; doi:10.3389/fimmu.2022.824586)
Supplement: Supplementary file 3 [file Table_1.doc]

**Supplementary Table 1. Detailed clinical information of patients subjected to IHC.**

| Patiens (*No.*) | Gender | Age (years) | Grade | Ki67(%) | Overall survival (days) | Survival status  (1:death, 0:survival) |
| --- | --- | --- | --- | --- | --- | --- |
| 1 | Male | 44 | II | 2 | 1082 | 0 |
| 2 | Female | 38 | II | 2 | 742 | 0 |
| 3 | Female | 47 | III | 15 | 923 | 0 |
| 4 | Female | 55 | III | 31 | 110 | 1 |
| 5 | Female | 63 | II | 5 | 400 | 1 |
| 6 | Male | 33 | III | 15 | 641 | 0 |
| 7 | Male | 46 | II | 1 | 881 | 1 |
| 8 | Male | 45 | III | 20 | 863 | 0 |
| 9 | Male | 31 | II | 2 | 566 | 0 |
| 10 | Male | 47 | II | 3 | 1060 | 0 |
| 11 | Male | 13 | III | 5-8 | 634 | 0 |
| 12 | Male | 48 | II | 5 | 44 | 0 |
| 13 | Male | 52 | II | 1 | 484 | 1 |
| 14 | Male | 40 | II | 5 | 959 | 0 |
| 15 | Male | 16 | III | 5 | 135 | 1 |
| 16 | Female | 47 | II | 2 | 1091 | 0 |
| 17 | Female | 35 | II | 5 | 1035 | 0 |
| 18 | Female | 44 | II | 2 | 1025 | 0 |
| 19 | Female | 43 | III | 10 | 148 | 0 |
| 20 | Female | 56 | III | 6 | 404 | 1 |
| 21 | Female | 50 | III | 23 | 564 | 1 |
| 22 | Female | 36 | II | 1 | 825 | 0 |
| 23 | Female | 48 | III | 40 | 82 | 1 |
| 24 | Female | 37 | III | 9 | 529 | 0 |
| 25 | Female | 14 | III | 1 | 474 | 0 |
| 26 | Female | 32 | III | 6 | 986 | 1 |
| 27 | Female | 34 | II | 2 | 86 | 1 |
| 28 | Female | 59 | III | 15 | 418 | 1 |
| 29 | Female | 61 | II | 5 | 874 | 0 |
| 30 | Female | 13 | II | 2 | 807 | 0 |

**Supplementary Table 2.** Detailed clinical information of patients subjected to IF, western blotting, qRT-PCR and ELISA.

| Patiens (*No.*) | Gender | Age (years) | Grade | Ki67 (%) | TNC mRNA level | Serum TNC concentration (ng/ml) |
| --- | --- | --- | --- | --- | --- | --- |
| 1 | Female | 46 | II | 5 | 3.420382 | 0.7857 |
| 2 | Male | 48 | II | 2 | 3.494648 | 2.563574 |
| 3 | Male | 67 | II | 6 | 5.016517 | 2.422205 |
| 4 | Female | 53 | II | 2 | 3.67232 | 0.490274 |
| 5 | Female | 53 | II | 5 | 3.767551 | 1.516563 |
| 6 | Male | 40 | II | 15 | 4.296573 | 3.129 |
| 7 | Male | 56 | II | 2 | 5.435134 | 4.636986 |
| 8 | Male | 44 | II | 3-4 | 4.1566 | 3.788772 |
| 9 | Female | 31 | II | 10 | 7.372145 | 6.569029 |
| 10 | Female | 36 | II | 5 | 6.668664 | 4.825478 |
| 11 | Male | 53 | II | 2 | 5.189985 | 4.07151 |
| 12 | Female | 53 | II | 5 | 5.749478 | 5.4852 |
| 13 | Female | 33 | II | 1 | 4.085514 | 3.977264 |
| 14 | Male | 32 | II | 3 | 3.653021 | 3.977264 |
| 15 | Male | 52 | II | 1 | 4.184529 | 1.694526 |
| 16 | Male | 65 | III | 20 | 3.341287 | 2.506034 |
| 17 | Female | 51 | III | 30 | 5.696137 | 21.130036 |
| 18 | Female | 43 | III | 15 | 6.558221 | 7.37012 |
| 19 | Male | 73 | III | 5 | 5.033971 | 2.519724 |
| 20 | Male | 52 | III | 15 | 3.90931 | 3.793136 |
| 21 | Male | 39 | III | 8 | 4.664638 | 6.050676 |
| 22 | Male | 48 | III | 20 | 4.761671 | 8.406826 |
| 23 | Female | 50 | III | 10 | 4.354273 | 6.144922 |
| 24 | Male | 66 | III | 15 | 6.16547 | 7.747104 |
| 25 | Female | 25 | III | 8 | 5.459131 | 5.506034 |
| 26 | Male | 15 | III | 12 | 5.567494 | 6.993136 |
| 27 | Male | 57 | III | 17 | 5.077913 | 8.218334 |
| 28 | Male | 48 | III | 10 | 6.32172 | 4.919724 |
| 29 | Female | 54 | III | 3-5 | 6.348844 | 6.521906 |
| 30 | Female | 42 | III | 15 | 5.70124 | 5.4852 |
| 31 | Male | 16 | III | 15 | 6.987607 | 5.218334 |
| 32 | Male | 29 | III | 40 | 8.166162 | 13.307618 |
| 33 | Male | 48 | Normal |  | 3.254971854 | 1.71536 |
| 34 | Male | 30 | Normal |  | 3.138334476 | 0.107424 |
| 35 | Male | 24 | Normal |  | 2.678008579 | 0.1207424 |
| 36 | Female | 52 | Normal |  | 2.34363018 | 0.207424 |
| 37 | Female | 33 | Normal |  | 3.459223217 | 0.584408 |
| 38 | Female | 27 | Normal |  | 3.445196919 | 0.38452 |

**Supplementary Table 3.** LGG patient information in the CGGA database

| CGGA (*n* = 441) | |
| --- | --- |
| **Grade** |  |
| II | 188 (43%) |
| III | 253 (57%) |
| **Gende**r |  |
| Female | 190 (43%) |
| Male | 251 (57%) |
| **Age (years)** |  |
| Median | 40 |
| Range | 11-72 |
| **Radio-status** |  |
| Treated | 313 (71%) |
| Un-treated | 102 (23%) |
| NA | 26 (6%) |
| **Chemo-status** |  |
| Treated | 283 (64%) |
| Un-treated | 132 (30%) |
| NA | 26 (6%) |
| **Survival time (days)** |  |
| Median | 1455 |
| Range | 51-5028 |
| **IDH mutation status** |  |
| Wild type | 96 (22%) |
| Mutant | 304 (69%) |
| NA | 41 (9%) |
| **1p19q codeletion status** |  |
| Codel | 130 (29%) |
| Non-codel | 272 (62%) |
| NA | 39 (9%) |
| **TNC expression（FPKM）** |  |
| Median | 13.33 |
| Range | 0.15-328.11 |
| **State of immune microenvironment** |  |
| Immunity-high | 61 (14%) |
| Immunity-low | 380 (86%) |
| **MGMT methylation status** |  |
| Methylated | 207 (47%) |
| Un-methylated | 134 (30%) |
| NA | 100 (23%) |

**Supplementary Table 4.** Sequence of qRT-PCR primers

| Name |  | Primer sequence (5'-3') |
| --- | --- | --- |
| β-actin | Forward | CATGTACGTTGCTATCCAGGC |
|  | Reverse | CTCCTTAATGTCACGCACGAT-3 |
| TNC | Forward | GCCCCTGATGTTAAGGAGCTG |
|  | Reverse | GGCCTCGAAGGTGACAGTT |
| CD4 | Forward | CAAGGAGGCAAAGGTCTCGAA |
|  | Reverse | CGGCACCTGACACAGAAGA |
| CD8 | Forward | ATGGCCTTACCAGTGACCG |
|  | Reverse | AGGTTCCAGGTCCGATCCAG |
| Iba1 | Forward | ATGAGCCAAACCAGGGATTTAC |
|  | Reverse | GGGATCGTCTAGGAATTGCTTGT |
| CD11b | Forward | GCCTTGACCTTATGTCATGGG |
|  | Reverse | CCTGTGCTGTAGTCGCACT |
| IL-10 | Forward | TCAAGGCGCATGTGAACTCC |
|  | Reverse | GATGTCAAACTCACTCATGGCT |
| TGF-β | Forward | CTAATGGTGGAAACCCACAACG |
|  | Reverse | TATCGCCAGGAATTGTTGCTG |
| PD-L1 | Forward | TGGCATTTGCTGAACGCATTT |
|  | Reverse | TGCAGCCAGGTCTAATTGTTTT |
| NKG2D | Forward | GAGTGATTTTTCAACACGATGGC |
|  | Reverse | ACAGTAACTTTCGGTCAAGGGAA |
