# Supplementary figures and images for: Tenascin-C can Serve as an Indicator for the Immunosuppressive Microenvironment of Diffuse Low-Grade Gliomas
Source: Front Immunol. 2022 Mar 16;13:824586. doi: 10.3389/fimmu.2022.824586 (PMC8966496; doi:10.3389/fimmu.2022.824586)

Figure. 3D

Merge

Iba-1

TNC

DAPI


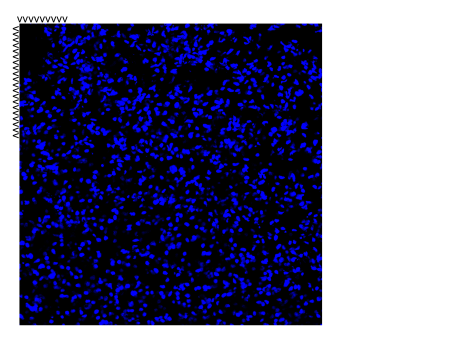

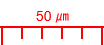

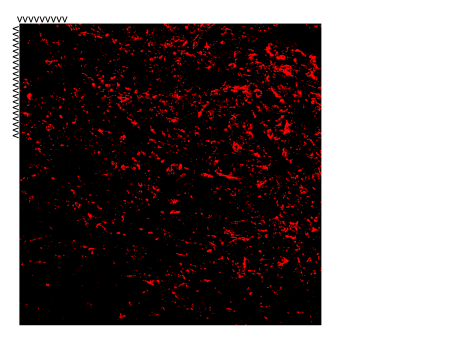

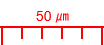

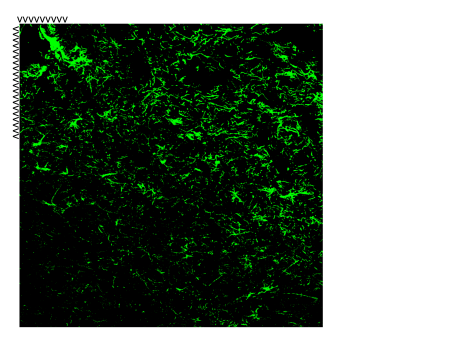

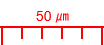

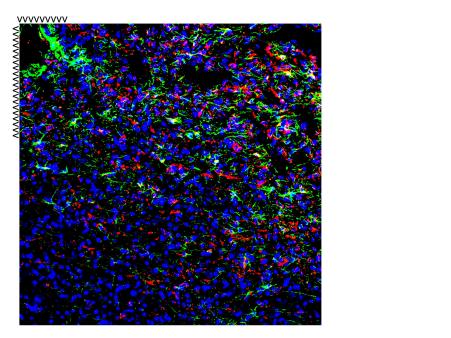

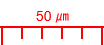


Merge

Iba-1

TNC

DAPI


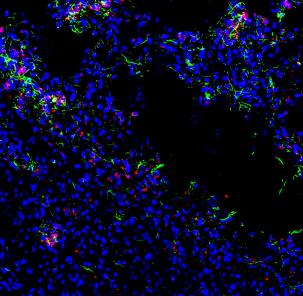

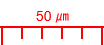

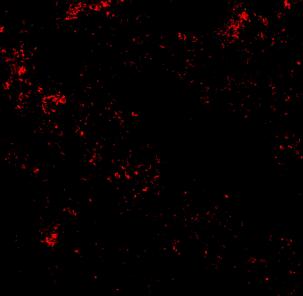

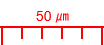

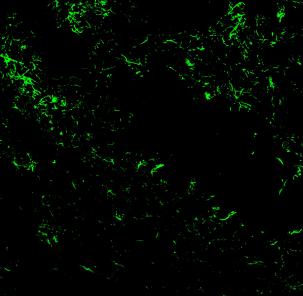

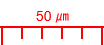

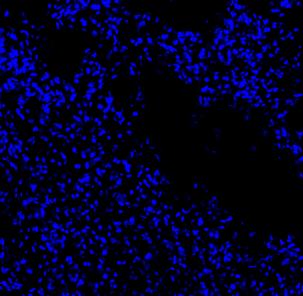

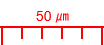


Merge

Iba-1

TNC

DAPI


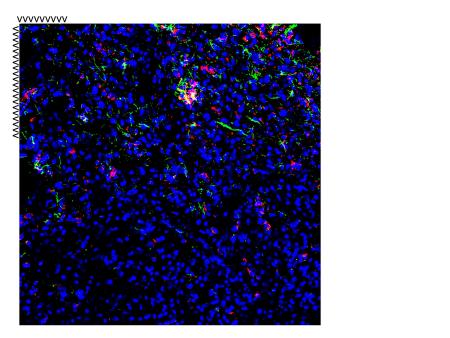

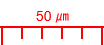

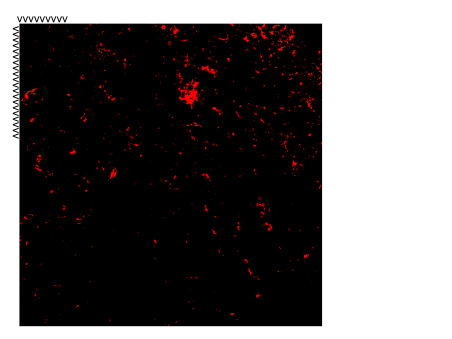

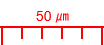

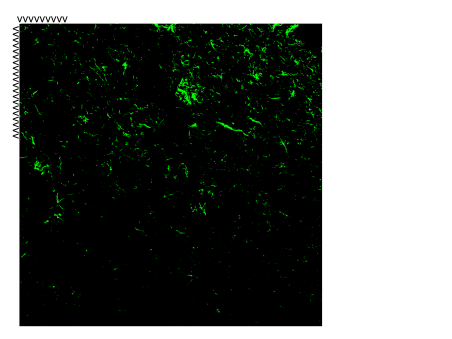

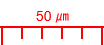

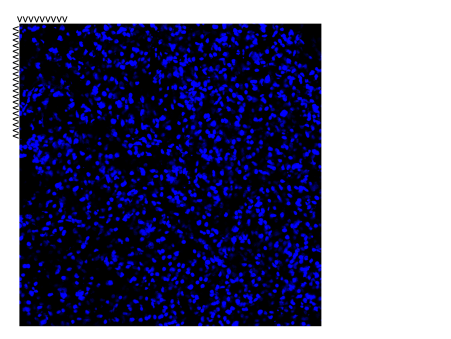

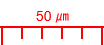


Figure. 4H

TNC

DAPI

Merge

CA9


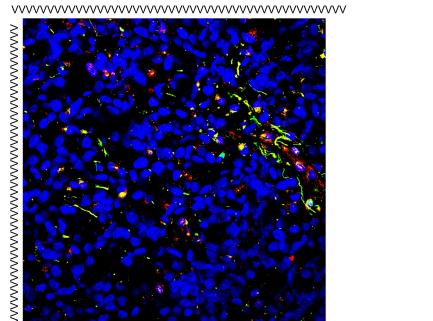

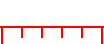

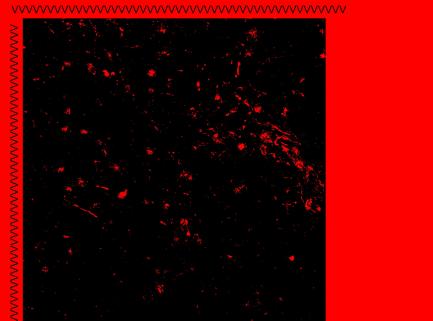

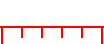

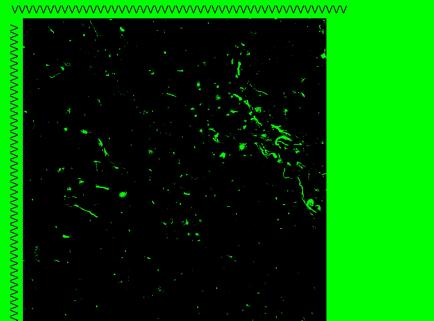

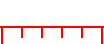

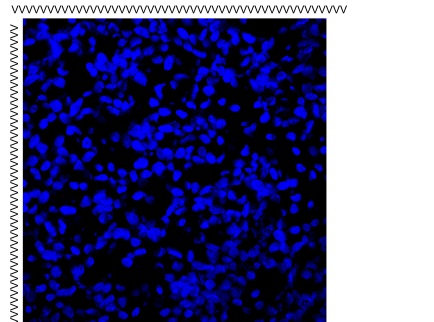

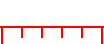

Supplement: Supplementary file 4 [file Table_2.docx]
