# Supplementary material for: Tenascin-C can Serve as an Indicator for the Immunosuppressive Microenvironment of Diffuse Low-Grade Gliomas
Source: Front Immunol. 2022 Mar 16;13:824586. doi: 10.3389/fimmu.2022.824586 (PMC8966496; doi:10.3389/fimmu.2022.824586)
Supplement: Supplementary file 5 [file Table_3.docx]

Figure. 2D (patient 1: TNC-Low)


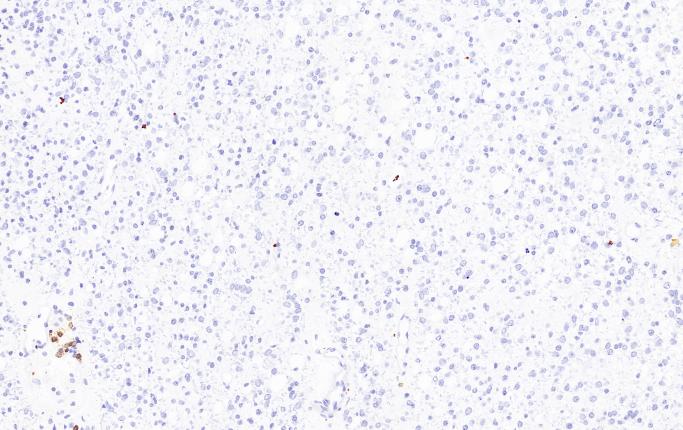

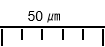

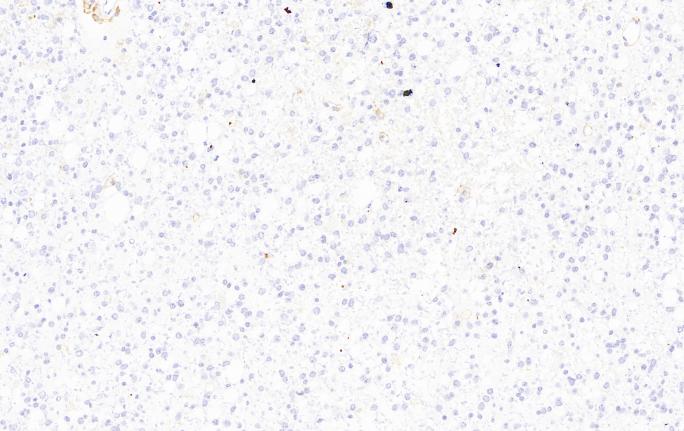

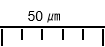

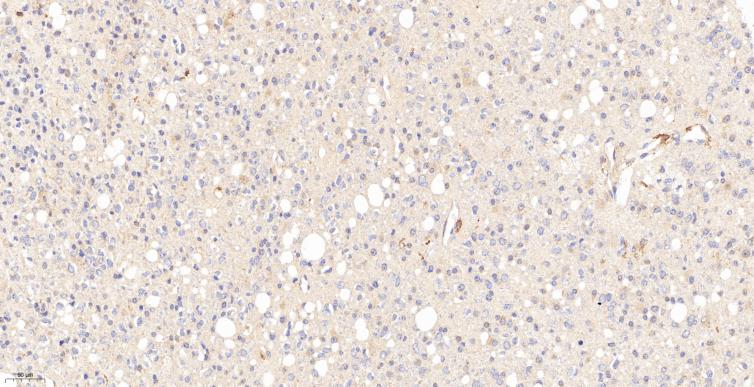

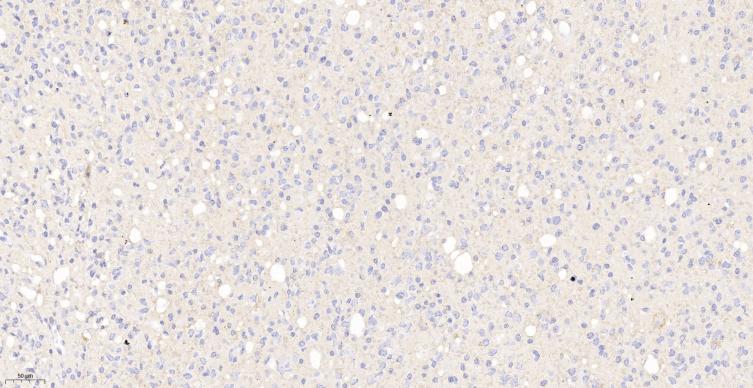


CD8

CD4

CD68

CD206

TNC


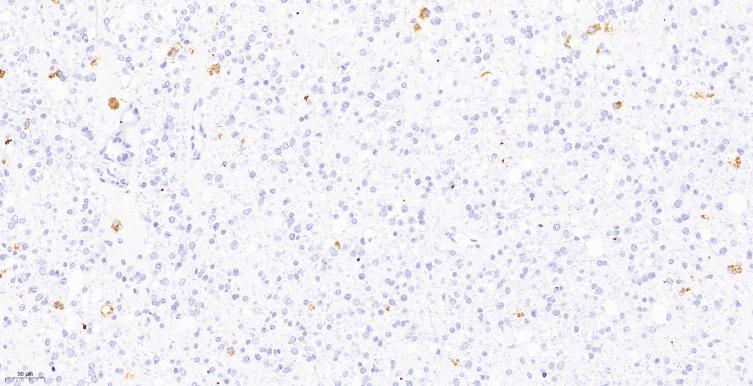


Figure. 2D (patient 2: TNC-High)

TNC

Figure. 2E (patient 3: TNC-Low)

CD8


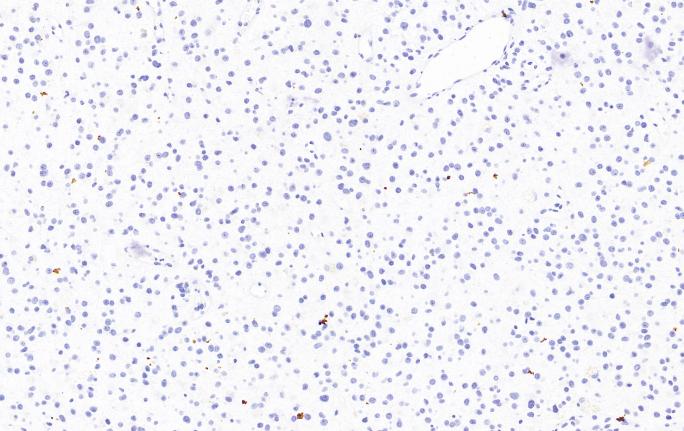

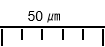


CD4


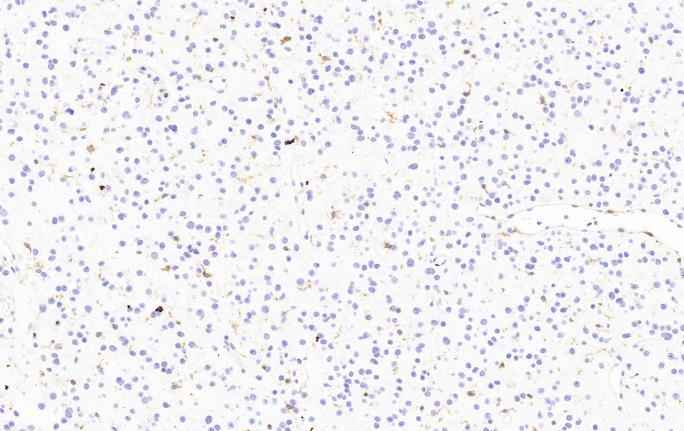

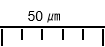

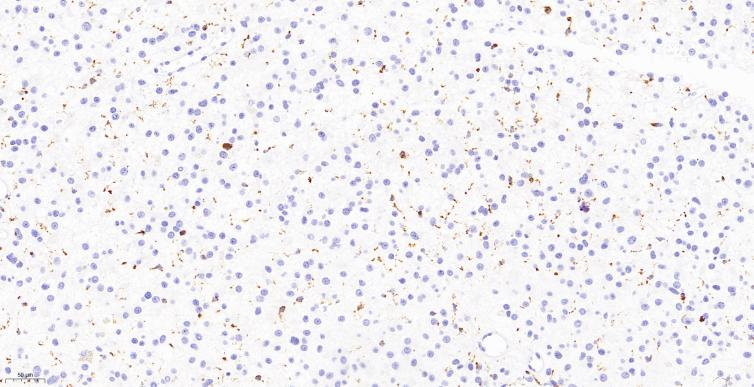


CD68

CD206


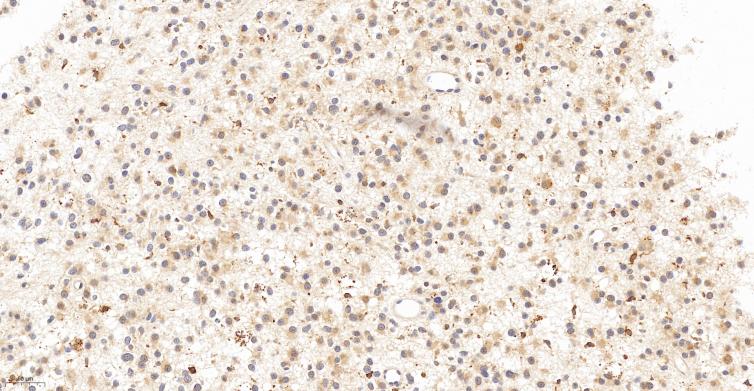

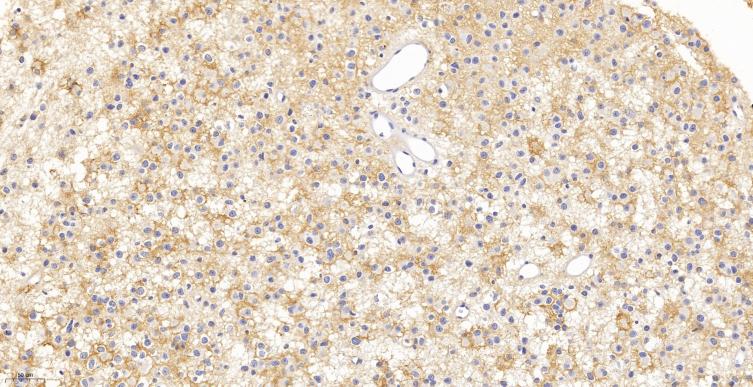


TNC


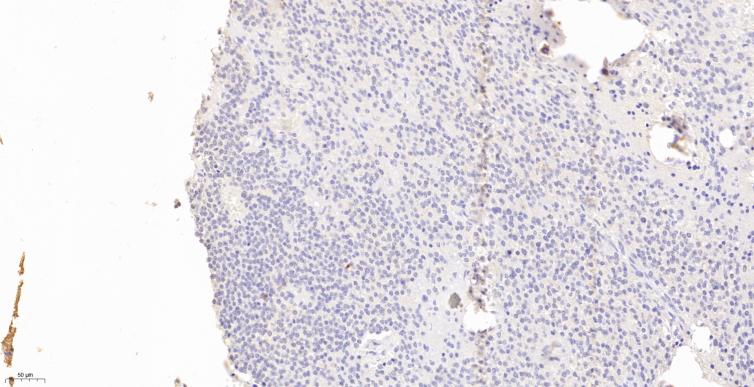


CD8


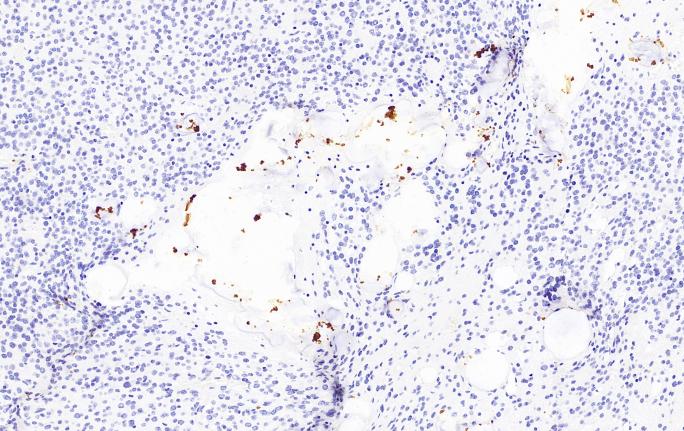

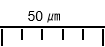


CD4


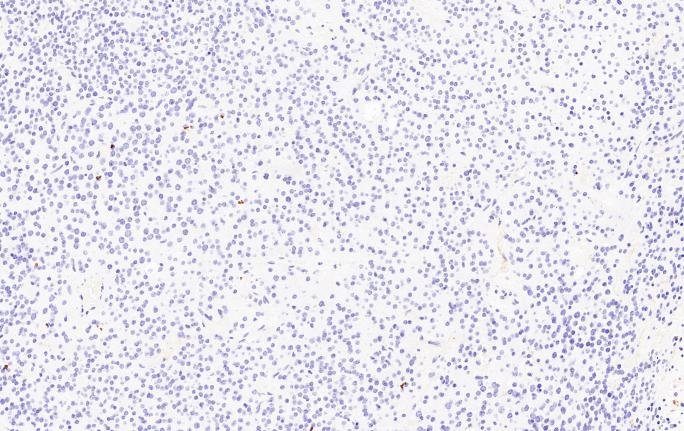

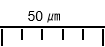


CD68


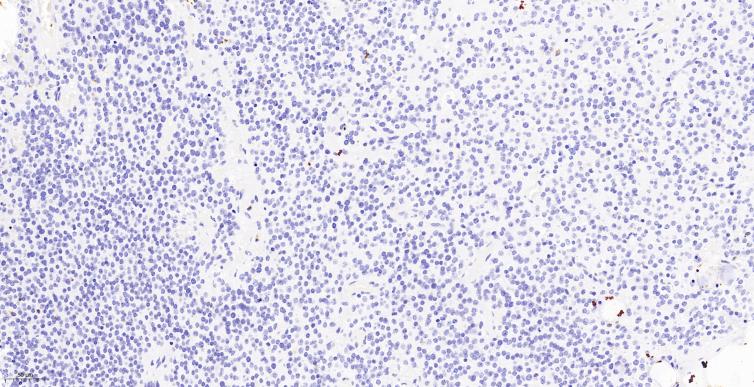


CD206


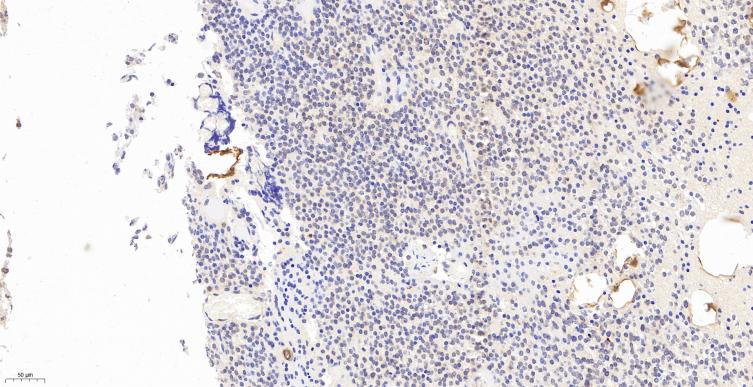


Figure. 2E (patient 4: TNC-High)

CD8


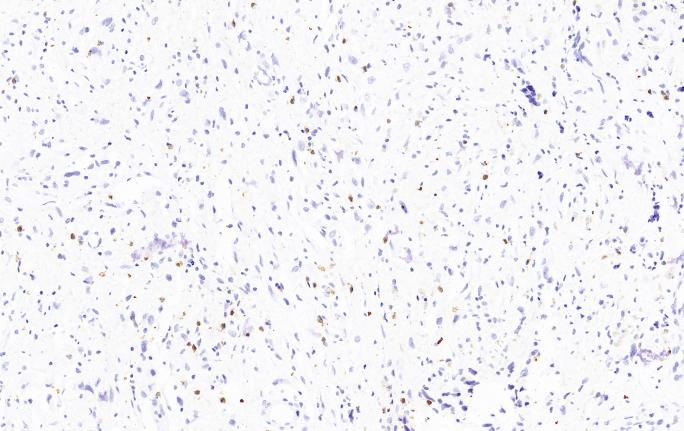

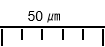


CD4


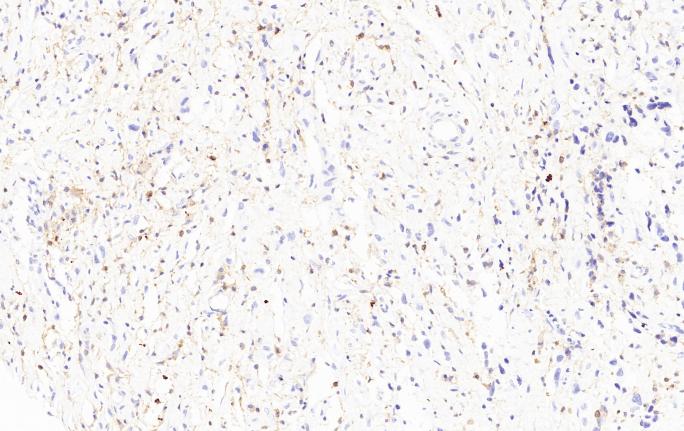

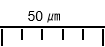

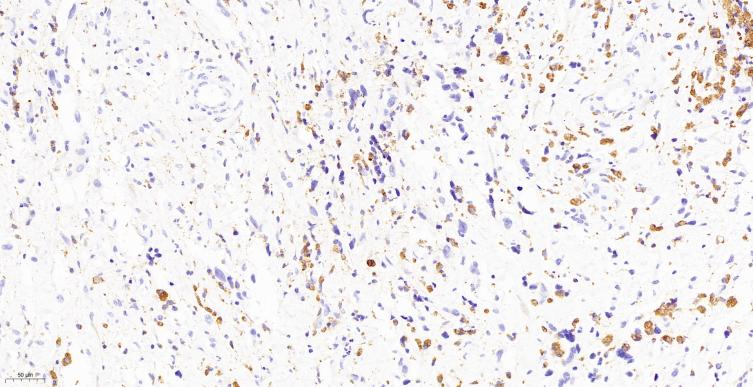


CD68

CD206

TNC


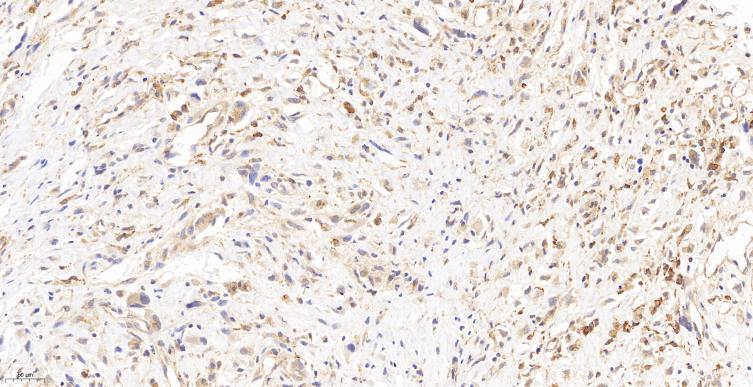

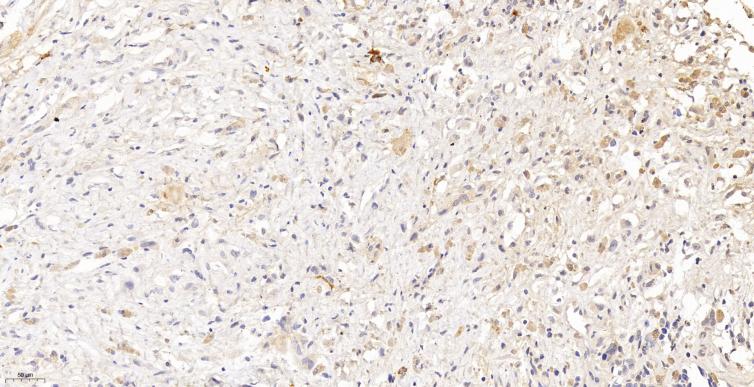


Figure. 4B (patient 1: TNC-Low)

FOXP3


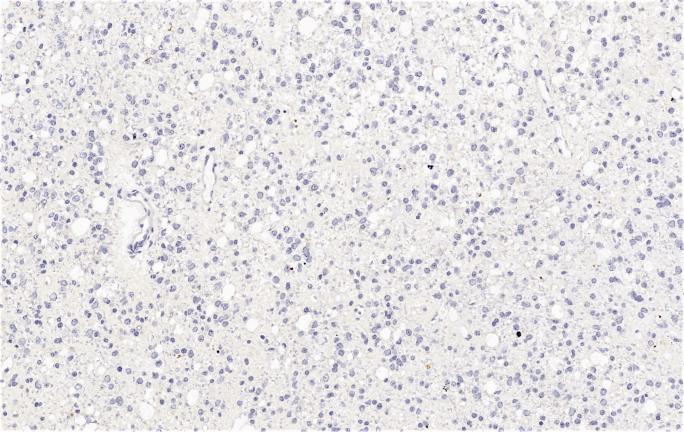

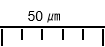


Figure. 4B (patient 2: TNC-High)

FOXP3


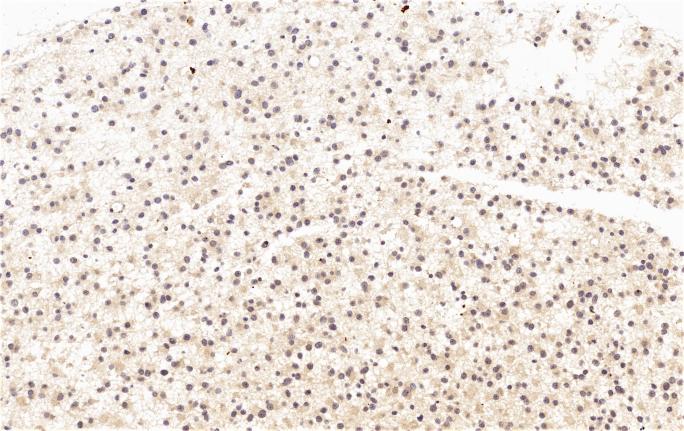

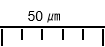


Figure. 4B (patient 3: TNC-Low)

FOXP3


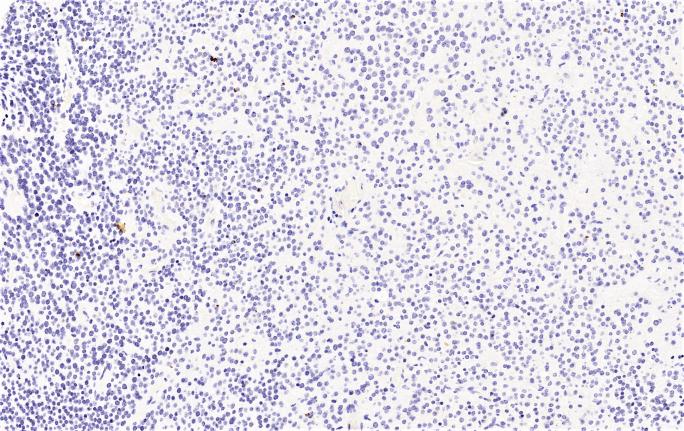

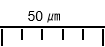


Figure. 4B (patient 4: TNC-High)

FOXP3


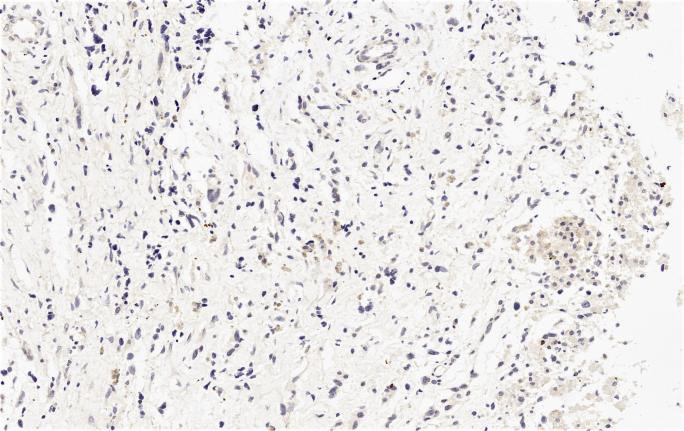

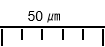


IHC Scores

| Patiens (No.) | CD68 | CD206 | CD4 | CD8 | FOXP3 | TNC |
| --- | --- | --- | --- | --- | --- | --- |
| 1 | 8.326 | 80.616 | 2.965 | 1.505 | 30.64 | 79.782 |
| 2 | 23.709 | 124.007 | 16.728 | 4.132 | 138.318 | 119.377 |
| 3 | 5.066 | 45.366 | 6.322 | 3.461 | 7.567 | 68.262 |
| 4 | 27.451 | 112.32 | 20.403 | 1.843 | 109.6 | 105.208 |
| 5 | 2.575 | 94.711 | 2.493 | 2.385 | 66.08 | 88.306 |
| 6 | 5.541 | 77.842 | 1.505 | 1.384 | 8.12 | 92.453 |
| 7 | 22.844 | 106.236 | 5.841 | 4.836 | 90.748 | 110.861 |
| 8 | 8.676 | 76.141 | 2.971 | 1.407 | 123.229 | 103.669 |
| 9 | 26.681 | 111.656 | 14.982 | 2.529 | 27.303 | 148.239 |
| 10 | 27.41 | 50.046 | 11.251 | 6.947 | 3.636 | 102.409 |
| 11 | 43.981 | 72.66 | 45.75 | 8.161 | 88.973 | 109.444 |
| 12 | 4.789 | 101.095 | 4.467 | 4.559 | 144.682 | 164.59 |
| 13 | 18.913 | 108.596 | 20.03 | 4.898 | 110.553 | 199.578 |
| 14 | 22.928 | 107.252 | 14.244 | 4.114 | 125.49 | 102.59 |
| 15 | 7.71 | 135.201 | 5.311 | 1.821 | 44.905 | 115.957 |
| 16 | 2.769 | 40.455 | 2.92 | 1.574 | 50.859 | 97.243 |
| 17 | 14.927 | 109.44 | 1.415 | 1.463 | 3.022 | 91.833 |
| 18 | 26.128 | 35.951 | 3.854 | 4.168 | 75.838 | 95.306 |
| 19 | 2.855 | 104.306 | 3.181 | 0.808 | 71.255 | 80.66 |
| 20 | 3.213 | 97.545 | 1.332 | 1.237 | 142.872 | 71.245 |
| 21 | 15.763 | 100.829 | 5.173 | 4.329 | 139.18 | 100.029 |
| 22 | 3.173 | 105.068 | 0.685 | 2.195 | 9.148 | 98.09 |
| 23 | 19.296 | 109.421 | 10.516 | 7.505 | 56.079 | 112.445 |
| 24 | 9.97 | 114.218 | 4.07 | 2.845 | 4.733 | 141.292 |
| 25 | 32.04 | 116.98 | 15.107 | 8.16 | 44.527 | 111.178 |
| 26 | 15.276 | 27.185 | 19.367 | 3.284 | 3.742 | 100.478 |
| 27 | 26.189 | 52.474 | 21.398 | 5.482 | 7.455 | 139.665 |
| 28 | 23.996 | 85.324 | 14.556 | 2.312 | 67.158 | 162.346 |
| 29 | 13.213 | 104.11 | 3.501 | 2.683 | 110.212 | 96.756 |
| 30 | 23.911 | 72.736 | 22.532 | 6.649 | 63.204 | 129.572 |
