# Supplementary material for: Tenascin-C can Serve as an Indicator for the Immunosuppressive Microenvironment of Diffuse Low-Grade Gliomas
Source: Front Immunol. 2022 Mar 16;13:824586. doi: 10.3389/fimmu.2022.824586 (PMC8966496; doi:10.3389/fimmu.2022.824586)
Supplement: Supplementary file 7 [file Table_5.docx]

**Western blotting**

Figure 2. G

TNC

TNC


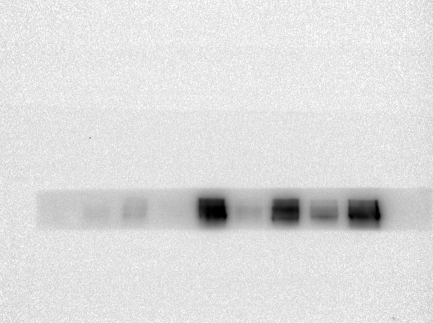

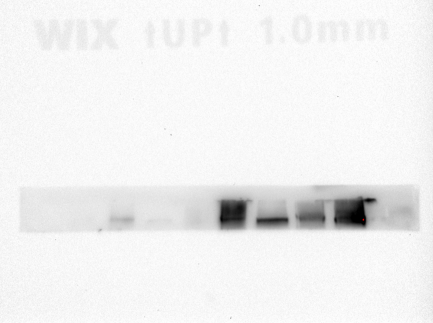


CD11b


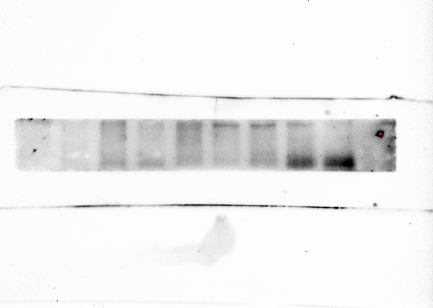

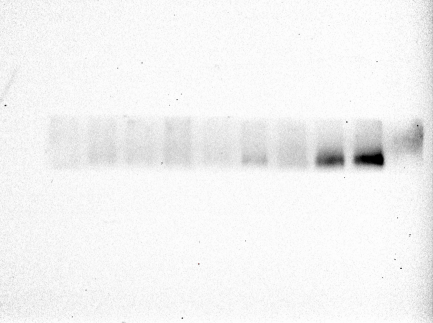


CD11b


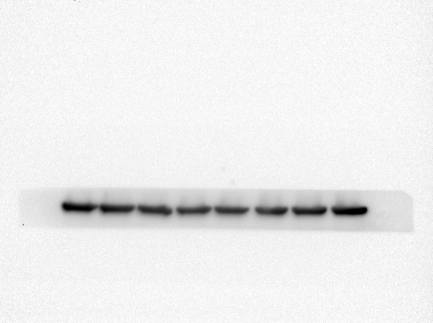

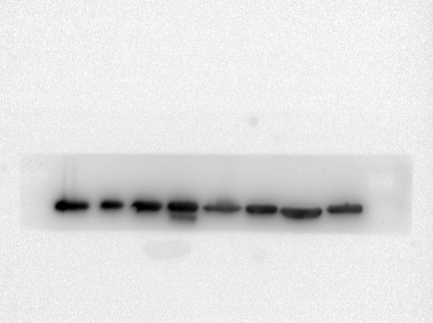


α-Tubulin

α-Tubulin

Figure 4. I

TNC

TNC


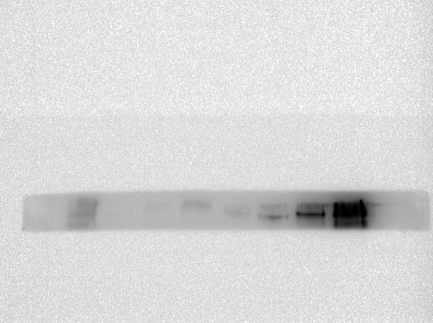

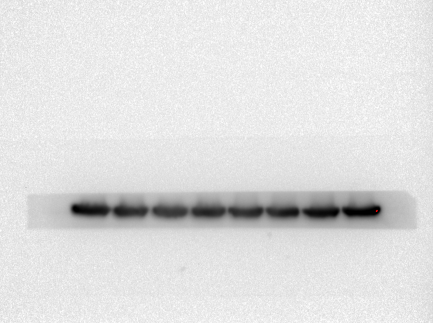

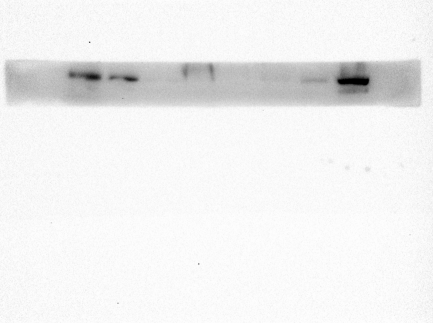

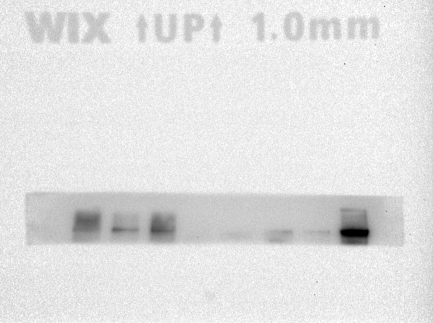

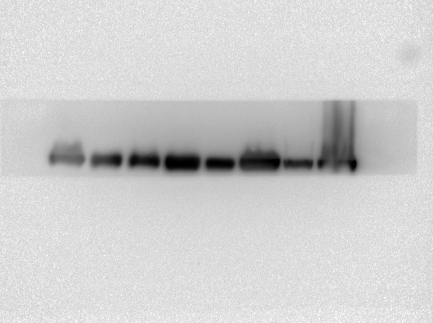

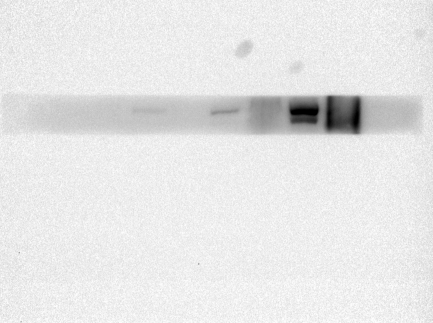


α-Tubulin

α-Tubulin

p-STAT3

p-STAT3

qRT-PCR

|  | TNC | CD8 | CD4 | IBA1 | TGF-β | IL-10 | CD11b | PD-L1 | NKD2G |
| --- | --- | --- | --- | --- | --- | --- | --- | --- | --- |
| 1 | 3.420382 | 4.911693 | 3.655633 | 3.64832 | 3.26987 | 3.460221 | 2.998372 | 2.681261 | 6.953272 |
| 2 | 3.494648 | 2.884835 | 3.125804 | 3.6587 | 3.15466 | 2.86237 | 3.907241 | 2.352366 | 7.52402 |
| 3 | 5.016517 | 3.26424 | 3.440033 | 3.35315 | 3.317804 | 2.510551 | 3.978068 | 2.410169 | 3.995016 |
| 4 | 3.67232 | 4.294882 | 3.012481 | 4.430604 | 5.262897 | 3.894411 | 3.528903 | 3.240271 | 4.736908 |
| 5 | 3.767551 | 3.547356 | 4.186713 | 4.153079 | 4.490621 | 3.640517 | 3.212602 | 2.841446 | 5.725732 |
| 6 | 4.296573 | 3.032846 | 2.834019 | 3.69745 | 4.21541 | 2.971001 | 3.568978 | 2.864426 | 2.180761 |
| 7 | 5.435134 | 4.630759 | 3.946114 | 4.250637 | 4.380884 | 3.076488 | 4.288765 | 3.411469 | 4.009339 |
| 8 | 4.1566 | 2.698205 | 2.638604 | 3.632485 | 5.21645 | 2.718385 | 3.978068 | 2.427282 | 4.081653 |
| 9 | 7.372145 | 4.401367 | 3.973381 | 4.182891 | 5.404914 | 4.15595 | 6.325784 | 3.707932 | 3.387742 |
| 10 | 6.668664 | 6.07118 | 4.751618 | 4.556548 | 5.230976 | 3.753209 | 3.763846 | 2.995598 | 6.884174 |
| 11 | 5.189985 | 6.98124 | 3.701373 | 4.25876 | 5.321844 | 3.755482 | 6.324842 | 3.678171 | 2.903128 |
| 12 | 5.749478 | 5.150607 | 5.073892 | 5.171027 | 7.636879 | 4.178686 | 4.786896 | 3.626138 | 2.762887 |
| 13 | 4.085514 | 4.602978 | 3.88553 | 4.797697 | 6.673404 | 3.771388 | 3.907241 | 3.127297 | 4.009339 |
| 14 | 3.653021 | 4.283578 | 4.06464 | 3.813756 | 4.224376 | 2.994922 | 3.365499 | 3.226614 | 6.953272 |
| 15 | 4.184529 | 5.094712 | 3.400647 | 4.6584 | 3.26841 | 3.532792 | 3.978068 | 2.941715 | 3.387742 |
| 16 | 3.341287 | 4.26182 | 3.428129 | 3.95641 | 3.32182 | 3.671028 | 3.212602 | 2.341927 | 6.409344 |
| 17 | 5.696137 | 5.611621 | 5.171171 | 4.510191 | 5.511469 | 4.744979 | 4.288757 | 3.207956 | 3.474506 |
| 18 | 6.558221 | 4.250688 | 3.371955 | 5.213647 | 7.62543 | 3.223823 | 5.786965 | 3.332574 | 3.044761 |
| 19 | 5.033971 | 4.082235 | 3.149336 | 4.154069 | 4.516032 | 4.04931 | 3.215879 | 2.884169 | 3.704448 |
| 20 | 3.90931 | 3.457277 | 4.498642 | 4.414067 | 4.891774 | 3.428364 | 2.998372 | 3.807431 | 3.474506 |
| 21 | 4.664638 | 3.880531 | 3.77472 | 3.790059 | 5.086106 | 3.417653 | 3.698994 | 2.748808 | 4.425306 |
| 22 | 4.761671 | 4.736908 | 3.995016 | 4.324732 | 3.561892 | 4.009339 | 5.812023 | 3.474506 | 6.409344 |
| 24 | 4.354273 | 4.623123 | 4.093273 | 4.241679 | 4.964583 | 4.172361 | 5.725732 | 3.826127 | 3.639813 |
| 24 | 6.16547 | 3.733966 | 4.455567 | 4.469827 | 5.018405 | 4.010152 | 4.796542 | 3.307269 | 3.995016 |
| 25 | 5.459131 | 6.356524 | 4.361116 | 4.368726 | 7.75461 | 4.234154 | 3.687931 | 3.518422 | 3.176145 |
| 26 | 5.567494 | 5.552497 | 3.911887 | 5.985664 | 6.58642 | 3.637867 | 5.321588 | 3.042467 | 2.574184 |
| 27 | 5.077913 | 3.637654 | 3.576098 | 4.115654 | 4.778254 | 3.592075 | 4.032159 | 3.550651 | 2.762887 |
| 28 | 6.32172 | 5.59392 | 5.128325 | 4.49829 | 5.546833 | 4.722033 | 6.974174 | 3.417665 | 4.736908 |
| 29 | 6.348844 | 3.70157 | 4.525153 | 3.756175 | 5.29532 | 4.684627 | 6.387451 | 2.937266 | 5.725732 |
| 30 | 5.70124 | 3.347048 | 3.645985 | 3.708071 | 3.63216 | 3.099112 | 6.369452 | 3.401735 | 3.602849 |
| 31 | 6.987607 | 3.978392 | 3.881685 | 3.794349 | 4.3472 | 3.469164 | 7.215469 | 3.725498 | 4.081653 |
| 32 | 8.166162 | 4.062385 | 4.863642 | 5.642346 | 5.065947 | 4.48682 | 5.663785 | 3.355794 | 2.180761 |
